# The prevalence of and variation in indicators of the quality and safety of long term aged care in Australia, 2019: a cross‐sectional population‐based study

**DOI:** 10.5694/mja2.52709

**Published:** 2025-06-24

**Authors:** Tesfahun C Eshetie, Gillian E Caughey, Catherine Lang, Olivia Ryan, Renuka Visvanathan, Craig Whitehead, Keith Evans, Janet K Sluggett, Jyoti Khadka, Carolyn Dawkins, Helena Williams, Miranda Starke, Sara Blunt, Anne Liddell, Megan Corlis, Anna Sheppeard, Penelope Lello, Marilyn von Thien, Steven L Wesselingh, Maria C Inacio

**Affiliations:** ^1^ Registry of Senior Australians (ROSA) South Australian Health and Medical Research Institute Adelaide SA; ^2^ Caring Futures Institute, Flinders University Adelaide SA; ^3^ University of South Australia Adelaide SA; ^4^ Adelaide Geriatrics Training and Research with Aged Care Centre University of Adelaide Adelaide SA; ^5^ Central Adelaide Local Health Network, SA Health Adelaide SA; ^6^ Southern Adelaide Local Health Network, SA Health Adelaide SA; ^7^ Flinders University Adelaide SA; ^8^ ECH Inc Adelaide SA; ^9^ SilverChain Group Limited Adelaide SA; ^10^ Council on the Ageing South Australia Adelaide SA; ^11^ James Brown Memorial Trust Adelaide SA; ^12^ Ageing Australia Adelaide SA; ^13^ Australian Nursing and Midwifery Federation (SA Branch) Adelaide SA; ^14^ South Australian Health and Medical Research Institute Adelaide SA

**Keywords:** Quality of health care, Aged, Health services for the aged, Aging, Benchmarking, Casemix, Health status indicators, Dementia, Healthcare disparities, Registries, Surveillance

## Abstract

**Objectives:**

To examine the prevalence of and variation in indicators of the quality and safety of care provided to older Australians who received subsidised long term care during 2019, by type of care (residential aged care or home care packages).

**Study design:**

Cross‐sectional population‐based study; analysis of linked data from the Registry of Senior Australians (ROSA) National Historical Cohort (National Aged Care Data Clearinghouse, National Death Index, Medicare Benefits Schedule, Pharmaceutical Benefits Scheme databases; South Australian, New South Wales, Victorian, and Queensland hospital admissions and emergency department [ED] presentations databases).

**Setting, participants:**

All people in the ROSA National Historical Cohort who received residential or home‐based aged care during the 2019 calendar year.

**Main outcome measures:**

Risk‐adjusted prevalence estimates (with 95% confidence intervals, CIs) for quality and safety indicators of care (twelve for residential care, fifteen for home care packages); proportions by indicator of outlier residential facilities and home care services (outside 95% CI for mean value) as a measure of variation in quality of care.

**Results:**

In 2019, 244 754 people received residential aged care in 2746 facilities; 149 104 people received home care packages through 2407 home care services. For residential aged care, indicator prevalence and variation were highest for antibiotic use (prevalence: 64.5% [95% CI, 64.3–64.7%]; 13.9% of facilities beyond upper 95% CI bound), high sedative load (prevalence: 45.2%, [95% CI, 45.0–45.4%]; 12.4% beyond upper 95% CI bound), and ED presentations (prevalence: 37.8% [95% CI, 37.6–38.0%]; 19.3% beyond upper 95% CI bound). For home care services, indicator prevalence and variation were highest for waiting time longer than six months (prevalence: 81.8% [95% CI, 81.4–82.1%]; 17.5% of services beyond upper 95% CI bound), ED presentations (prevalence: 43.2% [95% CI, 43.0–43.5%]; 14.7% beyond upper 95% CI bound), chronic disease management plans (prevalence: 43.2% [95% CI, 42.9–43.5%]; 12.9% below lower 95% CI bound), and home medicines reviews (prevalence: 3.2% [95% CI, 3.1–3.3%]; 28.9% below lower 95% CI bound). The proportions of home care recipients were larger than for facility residents for hospitalisations with delirium/dementia (home care, 10.5% [95% CI, 10.1–10.9%]; residents, 4.3% [95% CI, 4.2–4.4%]), weight loss/malnutrition (home care, 5.5% [95% CI, 5.3–5.6%]; residents, 2.5% [95% CI, 2.4–2.6%]), or medication‐related events (home care, 4.6% [95% CI, 4.5–4.7%]; residents, 2.4% [95% CI, 2.3–2.5%]).

**Conclusions:**

The marked national variations by residential or home aged care provider in antibiotic use, ED presentations, high sedative load, longer waiting times for home care services, home medicines reviews, and chronic disease management plans suggest these areas could benefit from targeted quality improvement strategies.

**The known**: The Registry of Senior Australians (ROSA) outcome monitoring system is a pragmatic system for monitoring and benchmarking the quality and safety of long term aged care in Australia.

**The new**: In 2019, variations in the quality of aged care between residential facilities and between home care services were quite marked, particularly with respect to indicators such as emergency department presentations, antibiotic use, high sedative load, waiting time for home care services, home medicines reviews, and chronic disease management plans.

**The implications**: Our findings could be used to identify areas of aged care that could be targeted by quality improvement programs.

During 2022–23, more than 565 000 people received government‐subsidised long term aged care in Australia: 250 273 in residential aged care and 314 971 who received home care packages.^1^ The need of our ageing population for high quality aged care services has long been unmet.^2^, ^3^ Quality and safety monitoring programs are critical for ensuring consistent high quality long term care.^4^ The approach, maturity, and impact of programs for monitoring the quality and safety of long term care differ between countries.^5^ In a recent review (to be published elsewhere), we identified nineteen residential care and thirteen home care programs, for twenty of which public reports are available.

In Australia, the National Mandatory Aged Care Quality Indicator Program (NMAC QI program) for residential aged care was introduced in 2019, but it does not cover home care.^6^ The NMAC QI program requires providers to report to the Department of Health and Aged Care on fourteen quality indicators in eleven domains every three months. Its reports describe differences by state and remoteness category and annual changes in the indicators. Five of the NMAC QI program indicators are included in the public reporting Star Ratings program.^6^, ^7^ We have raised concerns, however, about reporting only by aggregate facility level performance, not adjusting assessments for risk, and the provider reporting burden.^8^ Complementary programs for evaluating and informing the care of older people in long term care are needed.^8^ In the United States, at least five major organisations regularly monitor and report on the quality of hospital inpatient care, complementing the reports of the Centers for Medicare and Medicaid Services.^9^

In 2017, the Registry of Senior Australians (ROSA) was established to integrate population‐level health and aged care datasets from across Australia, facilitating the evaluation of the quality of aged care services. The ROSA outcome monitoring system, based on the ROSA data platform, is a pragmatic and low burden quality and safety monitoring and benchmarking system led by academics, clinicians, aged care providers, and aged care user representatives; it uses twelve risk‐adjusted quality and safety indicators for residential aged care, and fifteen for home care package services.^10^, ^11^, ^12^ The ROSA outcome monitoring system provides risk‐adjusted estimates that enable comparisons of care quality for aged care residential facilities and home care services, includes indicators that are not nationally monitored and indicator benchmarking information, and regularly assesses variation between providers in care quality. It can provide national evaluations of deidentified facilities and home care package services every two years (when new data are integrated) that can be used for population‐level evaluation of quality of care. In South Australia, annual facility and home care service reports are available to individual aged care providers.^10^ ROSA outcome monitoring system measures have been used by the Royal Commission into Aged Care Quality and Safety (2018–21)^13^, ^14^, ^15^, ^16^, ^17^ and in several national studies.^18^, ^19^

In this study, we used the ROSA outcome monitoring system to examine the prevalence of and variation in indicators of the quality and safety of care provided to older Australians who received subsidised long term care during 2019, by type of care (residential aged care or home care packages).

## Methods

We undertook a cross‐sectional population‐based study, analysing ROSA National Historical Cohort data.^10^ Briefly, the ROSA National Historical Cohort integrates deidentified data from national and state‐based aged care, health care, and social welfare datasets for older Australians who were assessed for subsidised aged care eligibility or who received aged care services during 1 January 2002 – 30 June 2020 (with data updates every two years). The included datasets are the Australian Institute of Health and Welfare National Aged Care Data Clearinghouse datasets, the National Death Index (NDI), the Australian Department of Social Services Data Over Multiple Individual Occurrences (DOMINO) database, the Australian Department of Health and Aged Care Medicare Benefits Schedule (MBS) and Pharmaceutical Benefits Scheme (PBS) databases, and South Australian, New South Wales, Victorian, and Queensland hospitalisations and emergency department (ED) presentations databases.^10^ For the study reported in this article, we analysed linked data from the National Aged Care Data Clearinghouse datasets and the NDI, MBS, PBS, and state‐level hospital and ED datasets.

Aged care services are subsidised by the Australian Department of Health and Aged Care. Residential aged care facilities provide accommodation, personal care, and nursing and general health care services.^20^ The Home Care Packages program provides a tailored, coordinated package of services to enable people to remain living at home.^21^

### Study cohort

We analysed data for all people aged 65 years or older (Aboriginal or Torres Strait Islander people: 50 years or older) who received subsidised long term aged care during the 2019 calendar year. Seven of the ROSA indicators relied on the hospital use data available only for four states (residential care: 213 446 people, 87.2% of people in cohort; home care: 129 852 people, 87.1% of people in cohort). We did not include Department of Veterans’ Affairs concession card holders for MBS‐based quality indicators — home medicines review and chronic disease management plans — as their access to MBS‐subsidised services is different.

### Outcomes

The twelve ROSA outcome monitoring system quality and safety indicators for residential and fifteen for home care include eight that are monitored in all states (antipsychotic use, chronic opioid use, high sedative load, antibiotic use, premature mortality; home care only: chronic disease management plan, home medicines review, waiting time for home care package services longer than six months) and seven that are monitored in the four states for which hospital use data are available (fall‐related hospitalisations, fractures, medication‐related hospitalisations, ED presentations, pressure injury‐related hospitalisations, weight loss or malnutrition‐related hospitalisations; people with dementia only: delirium or dementia‐related hospitalisations) (Supporting Information, tables 1 and 2).^11^, ^12^, ^22^ The three indicators for home care only were included following recommendations from the Royal Commission into Aged Care Quality and Safety.^11^

### Statistical analyses

We summarise as descriptive statistics the care recipient characteristics by type of care (residential or home care) and the number of facilities and home care services during 2019, nationally and for the four states for which hospitalisation‐related indicators can be assessed. For each indicator, we report estimated risk‐adjusted prevalence or incidence with the 95% confidence interval (CI). All indicator estimates were adjusted for age, sex (female or male), and number of health conditions, as well as for indicator‐specific covariates (eg, dementia, osteoporosis).^11^, ^12^, ^22^ The probability of a specific event (expected rate) was estimated using logistic regression models that included the relevant covariates. The risk‐adjusted rate was the ratio of the observed to expected probability multiplied by the national rate.

We report quality variation by facility or home care service level, and the proportion of indicator outlier values (outside the 95% CI for the mean value; potentially indicating suboptimal care), by long term care type. For chronic disease management plans and home medicines review, we deemed performance below the lower bound of the 95% CI to potentially indicate suboptimal care.

Facility or home care service level quality variations for each indicator were examined using funnel plots, stratified by facility or provider ownership type (private, not‐for‐profit, government), as care quality varies significantly by ownership type.^19^ The expected variation in quality was shown by the 95% CI for the indicator mean for facilities or home care services; the Wilson method for binomially distributed estimates was used to estimate CIs. Only facilities or home care services including twenty or more people are displayed in funnel plots to limit identifiability. All analyses were performed using SAS 9.4. We report our study according to the Reporting of studies conducted using observational routinely collected health data (RECORD) reporting checklist.^23^

### Ethics approval

The study was approved by the University of South Australia Human Research Ethics Committee (200489), the Australian Institute of Health and Welfare Ethics Committee (EO2022/4/1376), the South Australian Department for Health and Wellbeing Human Research Ethics Committee (HREC/18/SAH/90), and the New South Wales Population and Health Services Research Ethics Committee (2019/ETH12028).

## Results

In 2019, 244 754 people received residential aged care in 2746 facilities and 149 104 people received home care packages from 833 providers through 2407 home care services. Among residential facility residents, 159 758 (65.3%) were women, 135 571 (55.4%) had diagnoses of dementia, and their median age was 86 years (interquartile range [IQR], 80–91 years). Among home care recipients, 96 300 (64.6%) were women, 28 026 (18.8%) had diagnoses of dementia, and the median age was 83 years (IQR, 77–88 years) (Box 1).

Box 1 **Characteristics of older Australians who received subsidised long term aged care services during the 2019 calendar year, by care type**

**Table:** 

Characteristics	Residential care	Home care
Number of people	244 754	149 104
Number of facilities/services	2746	2407
People per facility or service, median (IQR)	79 (51–121)	32 (11–75)
Sex (women)	159 758 (65.3%)	96 300 (64.6%)
Age (years), median (IQR)	86 (80–91)	83 (77–88)
State		
New South Wales	81 126 (33.1%)	50 679 (34.0%)
Victoria	64 777 (26.5%)	37 965 (25.5%)
Queensland	46 381 (19.0%)	30 604 (20.5%)
South Australia	21 456 (8.8%)	10 604 (7.1%)
Other	31 014 (12.7%)	19 252 (12.9%)
Health conditions, median (IQR)	5 (3–7)	5 (3–7)
Dementia	135 571 (55.4%)	28 026 (18.8%)
Remoteness^24^		
Major cities	170 959 (69.8%)	102 677 (68.9%)
Inner regional	53 352 (21.8%)	35 552 (23.8%)
Outer regional	18 441 (7.5%)	9419 (6.3%)
Remote or very remote	2002 (0.9%)	1400 (0.9%)
Missing data	0	56 (< 0.1%)
Facility/service ownership		
Government	9757 (4.0%)	9603 (6.4%)
Not‐for‐profit	138 386 (56.5%)	105 768 (70.9%)
Private	96 611 (39.5%)	33 733 (22.6%)

IQR = interquartile range.

### National indicators (eight indicators)

The national proportions of people with medication‐related indicators were larger for facility residents than home care recipients: antibiotic use (residents, 64.5% [95% CI, 64.3–64.7%]; home care, 57.3% [95% CI, 57.1–57.6%]), high sedative load (residents: 45.2% [95% CI, 45.0–45.4%]; home care: 29.8% [95% CI, 29.6–30.1%]), chronic opioid use (residents: 26.1% [95% CI, 25.9–26.3%]; home care: 15.4% [95% CI, 15.2–15.6%]), and antipsychotic use (residents: 21.4% [95% CI 21.3–21.6%]; home care: 7.0% [95% CI, 6.9–7.2%]). The national premature mortality proportion was also larger for aged care facility residents (0.7% [95% CI, 0.6–0.7%]) than for those who received home care (0.3% [95% CI, 0.2–0.3%]) (Box 2).

The proportions of outliers (beyond the upper 95% CI bound) were larger for residential facilities than home care services for antibiotic use (facilities, 368 of 2638 [13.9%]; services, 118 of 1507 [7.8%]), high sedative load (facilities, 322 of 2606 [12.4%]; services, 57 of 1488 [3.8%]), chronic opioid use (facilities, 272 of 2628 [10.4%]; services, 85 of 1492 [5.7%]), and antipsychotic use (facilities, 176 of 2617 [6.7%]; services, 13 of 1502 [0.9%]) (Box 3, Box 4).

For home care‐only indicators, 81.8% (95% CI, 81.4–82.1%) of people waited more than six months for services, 43.2% (95% CI, 42.9–43.5%) received chronic disease management plans, and 3.2% (95% CI, 3.1–3.3%) had home medicines reviews (Box 2). The estimated proportions of care services below the lower 95% CI bound were 179 of 1388 (12.9%) for chronic disease management plans and 427 of 1477 for home medicines reviews (28.9%) (Box 3).

### State‐based indicators (seven indicators)

The proportions of home care recipients who received hospital care were larger than for facility residents for ED presentations (home care, 43.2% [95% CI, 43.0–43.5%]; residents, 37.8% [95% CI, 37.6–38.0%]) and hospitalisations with delirium/dementia (home care, 10.5% [95% CI, 10.1–10.9%]; residents, 4.3% [95% CI, 4.2–4.4%]), weight loss/malnutrition (home care, 5.5% [95% CI, 5.3–5.6%]; residents, 2.5% [95% CI, 2.4–2.5%]), or medication‐related events (home care, 4.6% [95% CI, 4.5–4.7%]; residents, 2.4% [95% CI, 2.3–2.5%]). The proportion of facility residents with fall‐related hospitalisations was larger than for home care recipients (residents, 13.6% [95% CI, 13.5–13.7%]; home care, 12.3% [95% CI, 12.1–12.5%]). The proportions of facility residents and home care recipients was similar for fractures (residents, 5.5% [95% CI, 5.4–5.6%]; home care, 5.4% [95% CI, 5.3–5.5%]) and pressure injury‐related hospitalisations (residents, 3.4% [95% CI, 3.3–3.5%]; home care, 3.5% [95% CI, 3.4–3.6%]) (Box 2).

The proportions of outliers (beyond the upper 95% CI bound) were larger for residential facilities than home care services for ED presentations (facilities, 442 of 2295 [19.3%]; services, 188 of 1276 [14.7%]), falls (facilities, 203 of 2290 [8.9%]; services, 60 of 1276 [4.7%]), pressure injury‐related hospitalisations (facilities, 79 of 2295 [3.4%]; services, 30 of 1276 [2.4%]), fractures (facilities, 42 of 2290 [1.8%]; services, 12 of 1276 [0.9%]), and delirium/dementia‐related hospitalisation (facilities, 29 of 1998 [1.5%]; services, two of 350 [0.6%]). The proportion of outliers (beyond upper 95% CI bound) was larger for home care services than for residential facilities for weight loss/malnutrition‐related hospitalisations (services, 41 of 1264 [3.2%]; facilities, 50 of 2287 [2.2%]) (Box 3, Box 4).

Box 2 Registry of Senior Australians outcome monitoring system quality and safety indicators, 2019: adjusted prevalence* (with 95% confidence intervals) by long term aged care type^†^

**Table:** 

	Residential care			Home care		
Indicator	Denominator	Numerator	Adjusted proportion	Denominator	Numerator	Adjusted proportion
**Australia**						
Number of people			244 419^‡^			149 104
Antibiotics	244 419	157 672	64.5% (64.3–64.7%)	149 104	85 502	57.3% (57.1–57.6%)
High sedative load	224 622^§^	101 616	45.2% (45.0–45.4%)	142 590^§^	42 530	29.8% (29.6–30.1%)
Chronic opioid use	236 989^§^	61 884	26.1% (25.9–26.3%)	144 442^§^	22 293	15.4% (15.2–15.6%)
Antipsychotics	231 862^§^	49 731	21.4% (21.3–21.6%)	147 224^§^	10 358	7.0% (6.9–7.2%)
Premature mortality	244 419	1639	0.7% (0.6–0.7%)	149 104	373	0.3% (0.2–0.3%)
Waiting time longer than six months for home care package services^¶^			—	45544^§^	37 233	81.8% (81.4–82.1%)
Chronic disease management plan^¶^			—	123 405^§^	53 291	43.2% (42.9–43.5%)
Home medicines review^¶^			—	141 749^§^	4526	3.2% (3.1–3.3%)
**New South Wales, Victoria, Queensland, South Australia**						
Number of people			213 446^‡^			129 852
Emergency department presentations	213 446	15 009	37.8% (37.6–38.0%)	129 852	56 155	43.2% (43.0–43.5%)
Delirium or dementia‐related hospitalisations	117 472^§^	5010	4.3% (4.2–4.4%)	23 833^§^	2502	10.5% (10.1–10.9%)
Weight loss or malnutrition‐related hospitalisations	206 975^§^	5129	2.5% (2.4–2.5%)	125 808^§^	6861	5.5% (5.3–5.6%)
Medication‐related hospitalisations	213 446	5117	2.4% (2.3–2.5%)	129 852	5997	4.6% (4.5–4.7%)
Pressure injury‐related hospitalisations	213 446	7308	3.4% (3.3–3.5%)	129 852	4582	3.5% (3.4–3.6%)
Fall‐related hospitalisations	213 446	28 962	13.6% (13.5–13.7%)	129 852	15 982	12.3% (12.1–12.5%)
Fractures	213 446	11 782	5.5% (5.4–5.6%)	129 852	7003	5.4% (5.3–5.5%)

* Adjusted for covariates listed in the Supporting Information, tables 1 and 2.

† The adjusted prevalence of indicators by care type and state are included in the Supporting Information, table 3.

‡ Multiple residential aged care episodes for the same resident and facility are combined.

§ After indicator‐specific exclusions (Supporting Information, tables 1 and 2).

¶ Home care only indicators.

Box 3 **Number and proportions of aged care residential facilities or home care services (used by twenty or more people in the ROSA National Historical Cohort) with indicator values outside the 95% confidence interval for the indicator, 2019, by type of long term care^†^**

**Table:** 

Indicator	Residential care			Home care		
	Facilities	Beyond upper 95% CI bound	Below lower 95% CI bound	Services	Beyond upper 95% CI bound	Below lower 95% CI bound
**National**						
Total number of facilities/services	2746	—	—	2407	—	—
Antibiotics	2638	368 (13.9%)	311 (11.8%)	1507	118 (7.8%)	109 (7.2%)
High sedative load	2606	322 (12.4%)	424 (16.3%)	1488	57 (3.8%)	139 (9.3%)
Chronic opioid use	2628	272 (10.4%)	413 (15.7%)	1492	85 (5.7%)	219 (14.7%)
Antipsychotics	2617	176 (6.7%)	372 (14.2%)	1502	13 (0.9%)	161 (10.7%)
Premature mortality	2638	3 (0.1%)	1517 (57.5%)	1507	1 (0.1%)	1234 (81.9%)
Waiting time for home care services^‡^	—	—	—	687	120 (17.5%)	67 (9.8%)
Chronic disease management plan^‡^	—	—	—	1388	140 (10.1%)	179 (12.9%)
Home medicines review^‡^	—	—	—	1477	21 (1.4%)	427 (28.9%)
**New South Wales, Victoria, Queensland, South Australia**						
Total number of facilities/services	2690	—	—	2030	—	—
Emergency department presentations	2295	442 (19.3%)	518 (22.6%)	1276	188 (14.7%)	188 (14.7%)
Delirium or dementia‐related hospitalisations	1998	29 (1.5%)	472 (23.6%)	350	2 (0.6%)	42 (12.0%)
Weight loss or malnutrition‐related hospitalisations	2287	50 (2.2%)	694 (30.3%)	1264	41 (3.2%)	250 (19.8%)
Medication‐related hospitalisations	2295	35 (1.5%)	607 (26.4%)	1276	20 (1.6%)	231 (18.1%)
Pressure injury‐related hospitalisations	2295	79 (3.4%)	592 (25.8%)	1276	30 (2.4%)	319 (25.0%)
Fall‐related hospitalisations	2290	203 (8.9%)	462 (20.2%)	1276	60 (4.7%)	172 (13.5%)
Fractures	2290	42 (1.8%)	320 (14.0%)	1276	12 (0.9%)	176 (13.8%)

CI = confidence interval.

† The numbers and proportions of outliers by care type and state are included in the Supporting Information, table 4.

‡ Home care only indicators.

Box 4 Distribution of adjusted antipsychotic use and emergency department presentation indicators* among aged care residential facilities and home care services (used by twenty people or more in the Registry of Senior Australians (ROSA) National Historical Cohort), 2019, by ownership type^†^

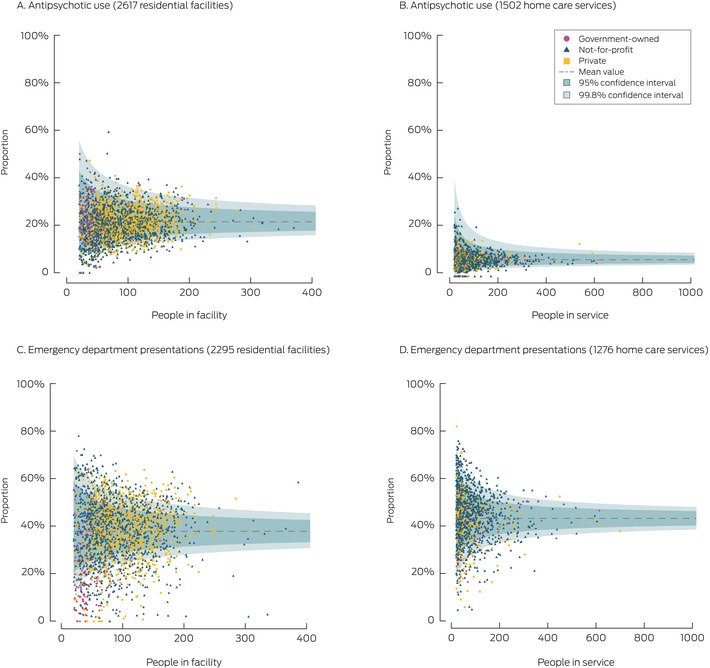

* Adjusted for covariates listed in the Supporting Information, tables 1 and 2.

† We selected one indicator each from the medication‐related and hospitalisation‐related indicators for this figure. The funnel plots for the indicators not shown here are included in the Supporting Information, figures 1 and 2.

## Discussion

In our analysis of integrated aged care and health care data, we found significant variation in the quality of care provided to long term care recipients in residential facilities or at home in Australia, with several low and high performing providers with respect to waiting time for services (home care only), ED presentations, antibiotic use, high sedative load, and home medicines reviews and chronic disease management plans (both home care only).

The prevalence of medication‐related indicators in facilities in 2019 was high: 21.4% of facility residents received antipsychotic medications, 45.2% had high sedative loads, 64.5% received antibiotics, and 26.1% had received opioid medications for longer periods. The estimated proportion of facility residents who received antipsychotics, based on population medication dispensing data, was consistent with NMAC QI program findings that about 20% of aged care facility residents in Australia received antipsychotics during July 2021 – December 2022,^25^ and was similar to the proportion reported for aged care facility residents in Canada (adjusted antipsychotic use without psychosis: 20.3%).^13^ In an earlier study, we found little change between 2014–2015 and 2018–2019 in the annual estimated national incidence of antibiotic, chronic opioid, or antipsychotic use or high sedative load among people in residential aged care.^26^ However, a recent United States study found that chronic opioid use by facility residents had declined from 14.1% in 2014 to 11.4% in 2018.^27^ While pain in aged care facility residents may often be missed or undertreated, with as many as 20% of people who experience pain not receiving analgesics,^28^ the prevalence of opioid prescribing is high; a systematic review found that at least 27% of aged care residents in Australia were dispensed at least one opioid for more than twelve months.^28^

The prevalence of medication‐related indicators among home care recipients in Australia was relatively stable during 2016–2019.^11^ Opioid (18% reduction, 2016‐17 to 2020‐21^29^) and antimicrobial medicines dispensing (9% reduction, 2013‐14 to 2016‐17^30^) declined among people of all ages living in the community while antipsychotic dispensing dropped by 11% among those aged 65 years and over) from 2016–17 to 2020–21.^31^ These changes contrast with our findings, suggesting differences in medication management and health care use between older Australians in general and those receiving residential or home‐based care.

We found that only 3.2% of home care recipients had government‐subsidised home medicines reviews and 43.2% received chronic disease management plans during 2019, two clinical interventions that can improve care and avert unnecessary hospitalisations.^32^, ^33^, ^34^, ^35^ Our findings are consistent with earlier assessments of the use of these services by people receiving home care and other older people.^30^, ^36^ However, national variation in the use of these services was quite substantial, suggesting that a nationally consistent and systematic approach to promoting them for people with home care packages is needed.^36^ The Department of Health and Aged Care has therefore announced changes to the chronic condition management plan (eg, a single general practitioner chronic condition management plan) to encourage regular reviews, which could reduce variation in quality of care.^37^

For the state‐based indicators, the estimated proportions of people in residential care admitted to hospital with pressure injuries (3.4%), falls (13.6%), or weight loss or malnutrition (2.5%) were smaller than those reported by the NMAC QI Program, whose estimates did not change markedly across the July 2021 – December 2022 reporting period: about 6% of residents had one or more pressure injuries, 2% falls with major injuries, and 9–11% had unplanned weight loss over three months.^25^ The prevalence of pressure injury‐related hospitalisation in our study was lower than reported by a study in eight European countries (6–13% within six months).^13^ The ROSA outcome monitoring system uses numbers of hospitalisations to measure these quality indicators, probably reflecting more severe events that require or are associated with hospitalisation. The estimated proportions of home care recipients admitted to hospital because of falls (12.3%) was lower than that reported for home care recipients in Canada (26.4%); for weight loss or malnutrition‐related hospitalisations our estimate (6%) was similar to that reported in Canada (7%).^13^ In our study, the proportions of home care recipients who presented to ED, were hospitalised because of weight loss or malnutrition, medication‐related events, or delirium and dementia were larger than for aged care facility residents. However, differences between care types in quality indicator performance should be interpreted cautiously, as differences in advanced care plans and care goals may contribute to variations in hospital use‐related indicators. Further, residual confounding after risk adjustment is possible, and understanding how care needs influence care quality outcome measures is important for improving individualised care.

We found considerable national variation in care quality among residential and home care providers in 2019. The three hospitalisation‐related indicators with the largest proportions of outlier facilities (upper 95% CI bound) were ED presentations, and falls‐ and pressure injury‐related hospitalisations. The variation in pressure injury‐related hospitalisations of facility residents was less marked than found by another Australian study,^38^ but the variation in ED presentations was similar to that reported in Ontario.^39^ Among the nationally assessable indicators, variability in the use of antibiotics and antipsychotics, and in high sedative load, is concerning; the proportion of outlier facilities with respect to antipsychotic and antibiotic use increased between 2016 and 2019, but those for high sedative load and chronic opioid use were stable.^12^ In contrast, a United States study found that variation in chronic opioid use in aged care homes increased by 16% between 2014 and 2018.^27^ Similar rates of antipsychotic prescribing variation in United States aged care facilities were reported in 2010.^40^ Significant variation in antibiotic use has also been reported in Europe and the United States.^41^

The marked national variations in antibiotic use, ED presentations, high sedative load, longer waiting times for home care services, home medicines reviews, and chronic disease management plans suggest these areas could benefit from targeted quality improvement strategies. Adherence to clinical care standards and clinical practice guidelines can promote high quality care and reduce care variation. For antibiotic use, adherence to antimicrobial stewardship guidelines can minimise inappropriate prescribing. Variation in high sedative load can be reduced by safe prescribing frameworks and adherence to appropriate psychotropic medicine use guidelines for aged care.^42^ For ED presentations, improving outreach and inreach multidisciplinary services, such as the 24‐hour on‐call services recommended by the Aged Care Royal Commission,^4^ could ensure timely care and urgent assessment and management.^4^ The new Support at Home program, which aims to reduce waiting time for services to less than three months and improve allied health and restorative care access, could help reduce variation in care quality.^43^

### Limitations

Our study evaluated the prevalence of and variations in indicators of quality of aged care in 2019, prior to the coronavirus disease 2019 (COVID‐19) pandemic and the Royal Commission into Aged Care Quality and Safety (2018–21) and subsequent national reforms. Integrating aged care and health care data is time‐intensive, typically requiring two to three years to obtain fully linked datasets from the various custodians for analysis. Although our access to more recent data has improved considerably, with full data linkage obtained in 2023, the inherent delay in data linkage remains and national improvement is required. For the hospitalisation‐related indicators, data were available from only four states, however they accounted for 87% of hospitalisations of aged care recipients during 2019.^10^ Data for admissions to private hospitals in South Australia were not available. However, 92% of emergency hospital admissions in Australia during 2017–18 were to public hospitals^44^ and private hospitals accounted for only 4% of all non‐admitted events during 2016–17.^45^ We probably underestimated certain indicators that rely on hospital use data for ascertainment, as only more severe cases would be recorded during the hospitalisation (eg, with weight loss/malnutrition). Clinical indications for medication‐ and service‐related indicators were not available. Further, our approach to identifying outliers has methodological limitations; for example, it probably identifies more lower outliers than when using a continuity correction. However, it is a conservative approach and unlikely to incorrectly identify providers with higher than expected rates.

### Conclusion

We found significant variations in quality measures of long term aged care for older people in Australia, particularly with regard to waiting time for home care services, ED presentations, antibiotic use, high sedative load, home medicines reviews, and chronic disease management plans. We also found differences in quality and variations in quality between residential and home‐based long term care. Our findings highlight national performance on key quality measures, and we have identified areas of long term aged care that could particularly benefit from targeted quality improvement strategies.

## Open access

Open access publishing facilitated by Flinders University, as part of the Wiley – Flinders University agreement via the Council of Australian University Librarians.

## Competing interests

Janet K Sluggett is a non‐executive director of Southern Cross Care SA, NT, VIC, and is a pharmacist accredited to perform comprehensive medication reviews.

## Data sharing

The data for this study were made available to the researchers under ethics, governance, and confidentiality agreements that do not allow public sharing.

## Supporting information


Supplementary methods and results
