# Supplementary material for: The prevalence of and variation in indicators of the quality and safety of long term aged care in Australia, 2019: a cross‐sectional population‐based study
Source: Med J Aust. 2025 Jun 24;223(4):189–96. doi: 10.5694/mja2.52709 (PMC12358104; doi:10.5694/mja2.52709)
Supplement: Supplementary file 1 — Supplementary methods and results [file MJA2-223-189-s001.pdf]

MJA
The Medical Journal of Australia

Supporting Information

Supplementary methods and results

This appendix was part of the submitted manuscript and has been peer reviewed.
It is posted as supplied by the authors.

Appendix to: Eshetie TC, Caughey GE, Lang C, et al. The prevalence of and variation in indicators of the quality and safety of long term aged care in Australia, 2019: a cross-sectional population-based study. *Med J Aust* 2025; doi: 10.5694/mja2.52709.

Supplementary methods

Table 1. Registry of Senior Australians (ROSA) Residential Care Outcome Monitoring System Quality and Safety Indicators Technical Specifications[1]

Indicator	Data Source[2]	Definition	Numerator	Denominator	Exclusions	Covariates
**National indicators (five indicators)**						
Antipsychotic use	PBS	Proportion of residents dispensed an antipsychotic	Number of permanent residents who have been dispensed at least one antipsychotic medication	Number of permanent residents	Residents with history of Schizophrenia, Huntington's disease	Age, sex, number of health conditions, antipsychotic use in the year prior to care entry
Chronic opioid use	PBS	Proportion of residents considered chronic opioid users	Number of permanent residents that are chronic opioid users*<br><br>*Chronic opioid use is defined as receiving any number of opioid medications for at least 90 days continuously, or for 120 non-consecutive days	Number of permanent residents	Residents receiving pharmacological cancer treatment or palliative care	Age, sex, number of health conditions (excluding pain)
High sedative load	PBS	Proportion of residents who experienced a high sedative load (sedative load >=3)	Number of permanent residents who experienced high sedative load (sedative load >=3) medication use within a 91-day period in a year	Number of permanent residents	Residents with history of Schizophrenia, Huntington's disease, receiving pharmacological cancer treatment or palliative care	Age, sex, number of health conditions
Antibiotic use	PBS	Proportion of residents dispensed an antibiotic	Number of permanent residents dispensed at least one antibiotic for systemic use	Number of permanent residents	None	Age, sex, number of health conditions
Premature mortality	NDI	Proportion of residents who had premature deaths	Number of permanent residents that had a cause of death recorded as 'external' and considered potentially avoidable, using ICD-10-AM	Number of permanent residents	None	Age, sex, number of health conditions
**State-based indicators (seven indicators)**						
Fall-related hospitalisation	SA APC, SA NAEC, NSW APDC, NSW	Proportion of residents who have experienced one or more falls resulting in hospitalisation	Number of permanent residents who had a fall that resulted in emergency department presentation, hospitalisation	Number of permanent residents	None	Age, sex, health conditions, dementia, level of mobility

	EDDC, VAED, VEMD, QLD EDC, QHAPDC, NDI	or emergency department presentation	(where onset is not during the hospitalisation), or death for fall			
Fractures	SA APC, SA NAEC, NSW APDC, NSW EDDC, VAED, VEMD, QLD EDC, QHAPDC, MBS, NDI	Proportion of residents who experienced at least one fracture	Number of permanent residents with an emergency department presentation or hospitalisation for fracture, or secondary diagnosis where onset is not during the hospitalisation, the external cause of the hospitalisation is fall, treatment for which MBS paid for, and deaths from fractures	Number of permanent residents	None	Age, sex, health conditions, level of mobility, dementia, osteoporosis
Medication-related hospitalisation	SA APC, SA NAEC, NSW APDC, NSW EDDC, VAED, VEMD, QLD EDC, QHAPDC	Proportion of residents who had an emergency department presentation or hospitalisation for or with medication-related events	Number of permanent residents who had an emergency department presentation or hospitalisation where medication-related event was recorded in any of the discharge diagnoses and was not identified as onset during hospitalisation or the external cause type for the encounter	Number of permanent residents	None	Age, sex, number of health conditions
Weight loss or malnutrition-related hospitalisation	SA APC, SA NAEC, NSW APDC, NSW EDDC, VAED, VEMD, QLD EDC, QHAPDC	Proportion of residents who had an emergency department presentation or hospitalisation, for or with weight loss or malnutrition	Number of residents who had an emergency department presentation or hospitalisation where malnutrition or weight loss was recorded in any of the discharge diagnoses for the encounter	Number of permanent residents	Pharmacological cancer treatment, palliative care	Age, sex, number of health conditions
Delirium or dementia-related hospitalisation	SA APC, SA NAEC, NSW APDC, NSW EDDC, VAED, VEMD, QLD EDC, QHAPDC	Proportion of residents who had an emergency department presentation or hospitalisation for delirium or dementia	Number of permanent residents who had an emergency department presentation or hospitalisation where dementia or delirium were the principal discharge diagnosis for the encounter	Number of residents living with dementia	None	Age, sex, number of health conditions (excluding dementia)
Emergency department presentation	SA APC, SA NAEC, NSW APDC, NSW EDDC, VAED,	Proportion of residents who had an emergency department presentation	Number of permanent residents who had an emergency department presentation	Number of permanent residents	None	Age, sex, number of health conditions, number of unplanned

	VEMD, QLD EDC, QHAPDC					hospitalisations in the year prior to care entry
Pressure injury-related hospitalisation	SA APC, SA NAEC, NSW APDC, NSW EDDC, VAED, VEMD, QLD EDC, QHAPDC	Proportion of residents who had an emergency department presentation or hospitalisation where pressure injury diagnoses were recorded	Number of permanent residents who had a hospitalisation or an emergency department presentation for or with a pressure injury	Number of permanent residents	None	Age, sex, number of health conditions

ACAP: Aged Care Assessment Program; **DVA**: Department of Veterans' Affairs; **HCP**: Home Care Packages; **ICD-10-AM**: International Classification of Diseases, Tenth Revision, Australian Modification; **MBS**: Medicare Benefits Schedule; **NDI**: National Death Index
NSAF: National Screening Assessment Form; **NSW APDC**: New South Wales Admitted Patient Data Collection; **NSW EDDC**: New South Wales Emergency Department Data Collection; **PBS**: Pharmaceutical Benefits Scheme; **QLD EDC**: Queensland Emergency Department Collection; **QHAPDC**: Queensland Hospital Admitted Patient Data Collection; **SA APC**: South Australian Admitted Patient Care; **SA NAEC**: South Australian Non-Admitted Emergency Care; **SAAS**: South Australian Ambulance Service; **VAED**: Victorian Admitted Episodes Dataset; **VEMD**: Victorian Emergency Minimum Dataset.

[1]Adapted from: Inacio MC, Lang C, Caughey GE, et al. The Registry of Senior Australians outcome monitoring system: quality and safety indicators for residential aged care. Int J Qual Health Care. 2020;32(8):502-510. doi:10.1093/intqhc/mzaa078.

[2]Refers to data sources used for the ascertainment of numerator definitions.

Table 2. Registry of Senior Australians (ROSA) Home Care Outcome Monitoring System Quality and Safety Indicators Technical Specifications[1]

Indicator	Data Source[2]	Definition	Numerator	Denominator	Exclusions	Covariates
**National Indicators (eight indicators)**						
Antipsychotic use	PBS	Proportion of home care recipients who were dispensed an antipsychotic	Number of home care recipients who were dispensed at least one antipsychotic	Number of home care recipients	Home care recipients with history of Schizophrenia, Huntington's disease	Age, sex, number of health conditions, antipsychotic use in the year prior to care entry
Chronic opioid use	PBS	Proportion of home care recipients who are chronic opioid users	Number of home care recipients who are chronic opioid users*<br><br>*Chronic opioid use is defined as receiving any number of opioid medications for at least 90 days continuously, or for 120 non-consecutive days	Number of home care recipients	Home care recipients receiving pharmacological cancer treatment	Age, sex, number of health conditions (excluding pain)
High sedative load	PBS	Proportion of home care recipients who experienced a high sedative load (sedative load ≥3)	Number of home care recipients who experienced high sedative load* (sedative load ≥3) medication use within a 91-day period in a year.<br><br>*Sedative load is calculated by summing the sedative rating of each medication dispensed during the same period	Number of home care recipients	Home care recipients with history of Schizophrenia, Huntington's disease or receiving pharmacological cancer treatment	Age, sex, number of health conditions
Antibiotic use	PBS	Proportion of home care recipients who were dispensed an antibiotic	Number of home care recipients who were dispensed at least one antibiotic for systemic use	Number of home care recipients	None	Age, sex, number of health conditions
Premature mortality	NDI	Proportion of home care recipients who died from premature causes, i.e., main cause of death is 'external' and considered potentially avoidable	Number of home care recipients who died and had a main cause of death recorded as 'external' and considered potentially avoidable, using ICD-10-AM	Number of home care recipients	None	Age, sex, number of health conditions
Chronic disease management plan	MBS	Proportion of home care recipients who had a chronic disease management plan	Number of home care recipients who had a Medicare subsidised chronic disease management plan	Number of home care recipients	DVA card holders	Age, sex, number of health conditions
Home medicines review	MBS	Proportion of home care recipients who had a home medicines review	Number of home care recipients who had a home medicines review	Number of home care recipients	DVA card holders	Age, sex, number of health conditions

Wait time for home care services	ACAP, NSAF, HCP episodes	Proportion of home care recipients who waited more than 6 months from aged care eligibility assessment approval to commencement of an HCP	Number of home care recipients who waited more than 6 months from aged care eligibility assessment approval to commencement of an HCP	Number of home care recipients who started home care for the first time	None	Age, sex, number of health conditions
**State-based indicators (seven indicators)**						
Fall-related hospitalisation	SA APC, SA NAEC, NSW APDC, NSW EDDC, VAED, VEMD, QLD EDC, QHAPDC NDI	Proportion of home care recipients who have experienced one or more falls resulting in hospitalisation or emergency department presentation	Number of home care recipients who had a fall that resulted in emergency department presentation, hospitalisation, or death for fall	Number of home care recipients	None	Age, sex, number of health conditions, dementia
Fractures	SA APC, SA NAEC, NSW APDC, NSW EDDC, VAED, VEMD, QLD EDC, QHAPDC, MBS, NDI	Proportion of home care recipients who had experienced at least one fracture	Number of home care recipients who had a fracture requiring an emergency department presentation or hospitalisation, or secondary diagnosis where onset is not during the hospitalisation, the external cause of the hospitalisation is fall, treatment for which MBS paid for, and deaths from fractures	Number of home care recipients	None	Age, sex, number of health conditions, dementia, osteoporosis
Medication-related hospitalisation	SA APC, SA NAEC, NSW APDC, NSW EDDC, VAED, VEMD, QLD EDC, QHAPDC	Proportion of home care recipients who had an emergency department presentation or hospitalisation for or with medication-related events	Number of home care recipients who had an emergency department presentation or hospitalisation where a medication-related event was recorded in any of the discharge diagnoses and was not identified as onset during hospitalisation or the external cause type for the encounter	Number of home care recipients	None	Age, sex, number of health conditions
Weight loss or malnutrition-related hospitalisation	SA APC, SA NAEC, NSW APDC, NSW EDDC, VAED, VEMD, QLD EDC, QHAPDC	Proportion of home care recipients who had an emergency department presentation or hospitalisation, for or with weight loss or malnutrition	Number of home care recipients who had an emergency department presentation or hospitalisation where malnutrition or weight loss was recorded in any of the discharge diagnoses for the encounter	Number of home care recipients	Home care recipients receiving pharmacological cancer treatment	Age, sex, number of health conditions

Delirium or dementia-related hospitalisation	SA APC, SA NAEC, NSW APDC, NSW EDDC, VAED, VEMD, QLD EDC, QHAPDC	Proportion of home care recipients with dementia who had an emergency department presentation or hospitalisation for delirium or dementia	Number of home care recipients with dementia who had an emergency department presentation or hospitalisation where dementia or delirium was the principal discharge diagnosis	Number of home care recipients with dementia	None	Age, sex, number of health conditions (excluding dementia)
Emergency department presentation	SA APC, SA NAEC, NSW APDC, NSW EDDC, VAED, VEMD, QLD EDC, QHAPDC	Proportion of home care recipients who had an emergency department presentation	Number of home care recipients who had an emergency department presentation	Number of home care recipients	None	Age, sex, number of health conditions, number of unplanned hospitalisations in the year prior to care entry
Pressure injury-related hospitalisation	SA APC, SA NAEC, NSW APDC, NSW EDDC, VAED, VEMD, QLD EDC, QHAPDC	Proportion of home care recipients who had an emergency department presentation or hospitalisation where pressure injury diagnoses were recorded	Number of home care recipients who had a hospitalisation or an emergency department presentation for or with a pressure injury (included in any of the diagnoses and not identified as onset during hospitalisation)	Number of home care recipients	None	Age, sex, number of health conditions

ACAP: Aged Care Assessment Program; **DVA**: Department of Veterans' Affairs; **HCP**: Home Care Packages; **ICD-10-AM**: International Classification of Diseases, Tenth Revision, Australian Modification; **MBS**: Medicare Benefits Schedule; **NDI**: National Death Index
NSAF: National Screening Assessment Form; **NSW APDC**: New South Wales Admitted Patient Data Collection; **NSW EDDC**: New South Wales Emergency Department Data Collection; **PBS**: Pharmaceutical Benefits Scheme; **QLD EDC**: Queensland Emergency Department Collection; **QHAPDC**: Queensland Hospital Admitted Patient Data Collection; **SA APC**: South Australian Admitted Patient Care; **SA NAEC**: South Australian Non-Admitted Emergency Care; **SAAS**: South Australian Ambulance Service; **VAED**: Victorian Admitted Episodes Dataset; **VEMD**: Victorian Emergency Minimum Dataset.

[1]Adapted from: Caughey GE, Lang CE, Bray SCE, et al. Quality and safety indicators for home care recipients in Australia: development and cross-sectional analyses. BMJ Open. 2022;12(8): e063152. doi:10.1136/bmjopen-2022-063152.

[2]Refers to data sources used for the ascertainment of numerator definitions.

Supplementary results

Table 3. Adjusted prevalence and 95% confidence intervals of ROSA Outcome Monitoring System quality and safety indicators, by type of long-term care and state, 2019

Indicator	Residential care				Home care			
	New South Wales	Victoria	Queensland	South Australia	New South Wales	Victoria	Queensland	South Australia
Antipsychotic use	20.9% (20.6-21.2%)	23.0% (22.7-23.4%)	20.6% (20.2-21.0%)	19.7% (19.2-20.3%)	6.7% (6.5-6.9%)	7.2% (7.0-7.5%)	7.2% (6.9-7.5%)	7.1% (6.6-7.6%)
Chronic opioid use	25.0% (24.7-25.3%)	25.7% (25.4-26.1%)	26.3% (25.9-26.7%)	27.4% (26.8-28.0%)	14.2% (13.9-14.5%)	14.3% (13.9-14.6%)	16.8% (16.4-17.2%)	17.3% (16.6-18.0%)
High sedative load	39.6% (39.2-39.9%)	49.9% (49.5-50.3%)	46.4% (45.9-46.9%)	47.4% (46.7-48.1%)	27.2% (26.8-27.6%)	30.7% (30.3-31.2%)	32.3% (31.7-32.8%)	31.1% (30.2-32.0%)
Antibiotic use	64.5% (64.2-64.9%)	64.8% (64.5-65.2%)	64.8% (64.3-65.2%)	63.7% (63.1-64.4%)	57.3% (56.9-57.7%)	57.1% (56.6-57.6%)	59.5% (58.9-60.0%)	55.9% (55.0-56.9%)
Fall-related hospitalisation	14.9% (14.6-15.1%)	11.6% (11.4-11.9%)	15.3% (15.0-15.7%)	11.1% (10.7-11.5%)	13.7% (13.4-14.0%)	9.8% (9.5-10.1%)	13.0% (12.6-13.4%)	12.5% (11.9-13.1%)
Fracture	5.5% (5.3-5.6%)	5.3% (5.1-5.5%)	6.3% (6.1-6.6%)	4.9% (4.6-5.2%)	5.5% (5.3-5.7%)	5.0% (4.8-5.2%)	5.6% (5.4-5.9%)	5.4% (5.0-5.9%)
Premature mortality	0.5% (0.5-0.6%)	0.8% (0.8-0.9%)	0.6% (0.6-0.7%)	0.7% (0.6-0.8%)	0.2% (0.2-0.3%)	0.2% (0.2-0.3%)	0.3% (0.2-0.3%)	0.2% (0.2-0.3%)
Medication-related hospitalisation	2.6% (2.5-2.7%)	1.9% (1.8-2.0%)	2.6% (2.4-2.7%)	2.7% (2.5-3.0%)	5.0% (4.8-5.2%)	3.9% (3.7-4.1%)	4.6% (4.4-4.9%)	5.4% (4.9-5.8%)
Weight loss or malnutrition-related hospitalisation	2.6% (2.5-2.7%)	1.9% (1.8-2.1%)	3.7% (3.5-3.8%)	1.2% (1.1-1.4%)	5.4% (5.2-5.6%)	5.1% (4.9-5.3%)	6.8% (6.5-7.1%)	3.2% (2.9-3.6%)
Delirium/dementia-related hospitalisation	3.8% (3.7-4.0%)	4.6% (4.4-4.8%)	4.7% (4.4-4.9%)	4.1% (3.8-4.5%)	9.9% (9.3-10.5%)	11.3% (10.5-12.1%)	10.8% (10.0-11.6%)	10.4% (9.1-11.8%)
Emergency department presentation	39.9% (39.6-40.2%)	34.4% (34.0-34.7%)	39.3% (38.8-39.7%)	37.5% (36.9-38.2%)	46.4% (46.0-46.9%)	40.4% (39.8-40.9%)	41.2% (40.7-41.8%)	44.5% (43.5-45.4%)
Pressure injury-related hospitalisation	3.7% (3.5-3.8%)	2.0% (1.9-2.1%)	5.2% (5.0-5.4%)	3.0% (2.8-3.2%)	3.6% (3.5-3.8%)	2.5% (2.4-2.7%)	4.8% (4.6-5.0%)	3.0% (2.7-3.4%)
Chronic disease management plan[a]	NA	NA	NA	NA	44.8% (44.3-45.2%)	42.4% (41.9-43.0%)	42.9% (42.3-43.5%)	44.3% (43.3-45.4%)
Home medicines review[a]	NA	NA	NA	NA	2.9% (2.8-3.1%)	3.7% (3.5-3.9%)	3.2% (3.0-3.4%)	3.3% (3.0-3.7%)

[a]Home care only quality and safety indicators; NA – not applicable.

Table 4. Number of facilities or home care services with indicators above and below 95% confidence intervals, by type of long-term care and state, 2019

Indicator/State	Residential care			Home care		
	Facilities included (≥20)	Above 95%CI	Below 95%CI	Services included (≥20)	Above 95%CI	Below 95%CI
Antipsychotic use						
New South Wales	862	49 (5.7%)	147 (17.2%)	494	3 (0.6%)	52 (10.5%)
Victoria	721	73 (10.1%)	67 (9.3%)	371	1 (0.3%)	29 (7.8%)
Queensland	456	28 (6.1%)	77 (16.9%)	319	4 (1.3%)	33 (10.3%)
South Australia	238	4 (1.7%)	45 (18.9%)	90	1 (1.1%)	4 (4.4%)
Chronic opioid use						
New South Wales	867	78 (9.0%)	189 (21.8%)	490	18 (3.7%)	99 (20.2%)
Victoria	726	69 (9.5%)	114 (15.7%)	368	21 (5.7%)	78 (21.2%)
Queensland	456	53 (11.6%)	65 (14.3%)	317	17 (5.4%)	21 (6.6%)
South Australia	238	21 (8.8%)	17 (7.1%)	89	6 (6.7%)	7 (7.9%)
High sedative load						
New South Wales	859	35 (4.1%)	296 (34.5%)	490	11 (2.2%)	76 (15.5%)
Victoria	716	150 (20.9%)	32 (4.5%)	368	14 (3.8%)	24 (6.5%)
Queensland	454	67 (14.8%)	56 (12.3%)	316	20 (6.3%)	17 (5.4%)
South Australia	238	24 (10.1%)	10 (4.2%)	89	4 (4.5%)	8 (9.0%)
Antibiotic use						
New South Wales	870	112 (12.9%)	95 (10.9%)	495	40 (8.1%)	34 (6.9%)
Victoria	729	93 (12.8%)	80 (11.0%)	371	31 (8.4%)	31 (8.4%)
Queensland	458	80 (17.5%)	55 (12.0%)	320	34 (10.6%)	16 (5.0%)
South Australia	238	34 (14.3%)	34 (14.3%)	90	3 (3.3%)	9 (10.0%)
Fall-related hospitalisation						
New South Wales	867	114 (13.1%)	134 (15.4%)	495	35 (7.1%)	44 (8.9%)
Victoria	728	21(2.9%)	188 (25.8%)	371	3 (0.8%)	87 (23.5%)
Queensland	457	61 (13.3%)	68 (14.9%)	320	18 (5.6%)	28 (8.8%)
South Australia	238	7 (2.9%)	72 (30.3%)	90	4 (4.4%)	13 (14.4%)
Fracture						
New South Wales	867	14 (1.6%)	116 (13.4%)	495	4 (0.8%)	67 (13.5%)
Victoria	728	9 (1.2%)	125 (17.1%)	371	4 (1.1%)	55 (14.8%)
Queensland	457	18 (3.9%)	45 (9.9%)	320	3 (0.9%)	44 (13.8%)
South Australia	238	1 (0.4%)	34 (14.2%)	90	1 (1.1%)	10 (11.1%)

Indicator/State	Residential care			Home care		
	Facilities included (≥20)	Above 95%CI	Below 95%CI	Services included (≥20)	Above 95%CI	Below 95%CI
Premature mortality						
New South Wales	870	1 (0.1%)	555 (63.8%)	495	1 (0.2%)	413 (83.4%)
Victoria	729	0 (0.0%)	372 (51.0%)	371	0 (0.0%)	304 (81.9%)
Queensland	458	1 (0.2%)	259 (56.6%)	320	0 (0.0%)	261 (81.6%)
South Australia	238	0 (0.0%)	134 (56.3%)	90	0 (0.0%)	71 (78.9%)
Medication-related hospitalisation						
New South Wales	870	22 (2.5%)	210 (24.1%)	495	12 (2.4%)	77 (15.6%)
Victoria	729	1 (0.1%)	228 (31.3%)	371	0 (0.0%)	90 (24.3%)
Queensland	458	8 (1.7%)	109 (23.8%)	320	3 (0.9%)	52 (16.3%)
South Australia	238	4 (1.7%)	60 (25.2%)	90	5 (5.6%)	90 (24.3%)
Weight loss or malnutrition-related hospitalisation						
New South Wales	867	27 (3.1%)	268 (30.9%)	490	21 (4.3%)	113 (23.1%)
Victoria	726	1 (0.1%)	254 (35.0%)	368	4 (1.1%)	69 (18.8%)
Queensland	456	22 (4.8%)	62 (13.6%)	317	15 (4.7%)	28 (8.8%)
South Australia	238	0 (0.0%)	110 (46.2%)	89	1 (1.1%)	40 (44.9%)
Delirium/dementia-related hospitalisation						
New South Wales	756	5 (0.7%)	200 (26.5%)	134	1 (0.7%)	23 (17.2%)
Victoria	617	13 (2.1%)	135 (21.9%)	83	1 (1.2%)	6 (7.2%)
Queensland	414	6 (1.5%)	91 (22.0%)	92	0 (0.0%)	7 (7.6%)
South Australia	211	5 (2.4%)	46 (21.8%)	41	0 (0.0%)	6 (14.6%)
Emergency department presentation						
New South Wales	870	226 (26.0%)	133 (15.3%)	495	115 (23.2%)	53 (10.5%)
Victoria	729	70 (9.6%)	225 (30.9%)	371	30 (8.1%)	69 (18.6%)
Queensland	458	109 (23.8%)	101 (22.1%)	320	26 (8.1%)	59 (18.4%)
South Australia	238	37 (15.5%)	59 (24.8%)	90	17 (18.9%)	7 (7.8%)
Pressure injury-related hospitalisation						
New South Wales	870	33 (3.8%)	186 (21.4%)	495	7 (1.4%)	115 (23.2%)
Victoria	729	1 (0.1%)	305 (41.8%)	371	2 (0.5%)	122 (32.9%)
Queensland	458	43 (9.4%)	35 (7.6%)	320	21 (6.6%)	54 (16.9%)
South Australia	238	2 (0.8%)	66 (27.7%)	90	0 (0.0%)	28 (31.1%)
Chronic disease management plan[a]						
New South Wales	NA	NA	NA	451	72 (16.0%)	47 (10.4%)
Victoria	NA	NA	NA	350	28 (8.0%)	42 (12.0%)

Indicator/State	Residential care			Home care		
	Facilities included (≥20)	Above 95%CI	Below 95%CI	Services included (≥20)	Above 95%CI	Below 95%CI
Queensland	NA	NA	NA	291	22 (7.6%)	38 (13.1%)
South Australia	NA	NA	NA	85	5 (5.9%)	9 (10.6%)
Home medicines review[a]						
New South Wales	NA	NA	NA	480	5 (1.0%)	165 (34.4%)
Victoria	NA	NA	NA	369	8 (2.2%)	77 (20.9%)
Queensland	NA	NA	NA	313	2 (0.6%)	94 (30.0%)
South Australia	NA	NA	NA	89	5 (5.6%)	24 (27.0%)
Wait-time for home care services[a]						
New South Wales	NA	NA	NA	208	29 (13.9%)	30 (14.4%)
Victoria	NA	NA	NA	161	26 (16.1%)	11 (6.8%)
Queensland	NA	NA	NA	162	33 (20.4%)	10 (6.2%)
South Australia	NA	NA	NA	64	19 (29.7%)	0 (0.0%)

[a]Home care only quality and safety indicators; NA - not applicable.

Figure 1. Funnel plots illustrating facility level variations in care, by ownership, 2019

Indicator 1: Adjusted high sedative load among residents of 2606 residential aged care facilities in Australia

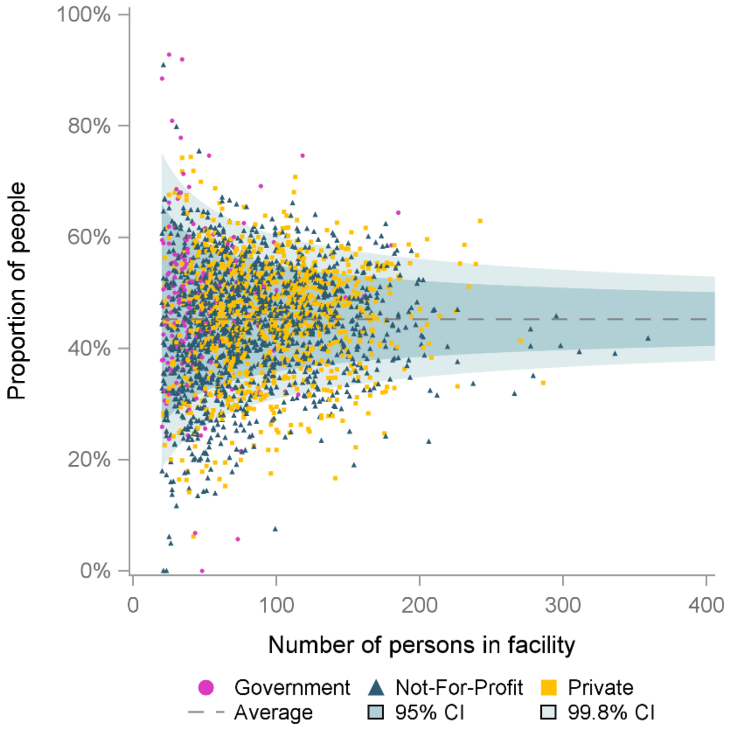

Indicator 2: Adjusted chronic opioid use among residents of 2628 residential aged care facilities in Australia

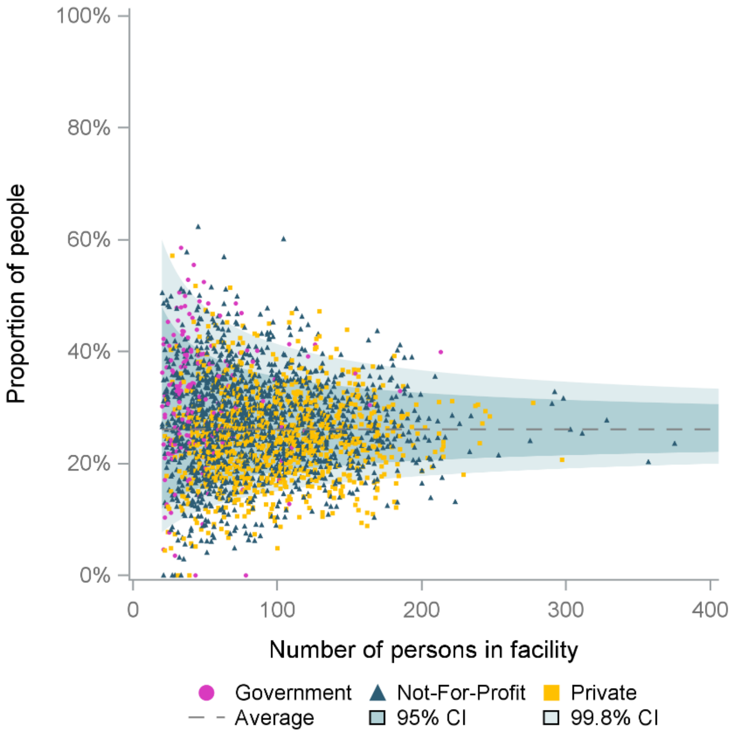

Indicator 3: Adjusted antibiotic use among residents of 2638 residential aged care facilities in Australia

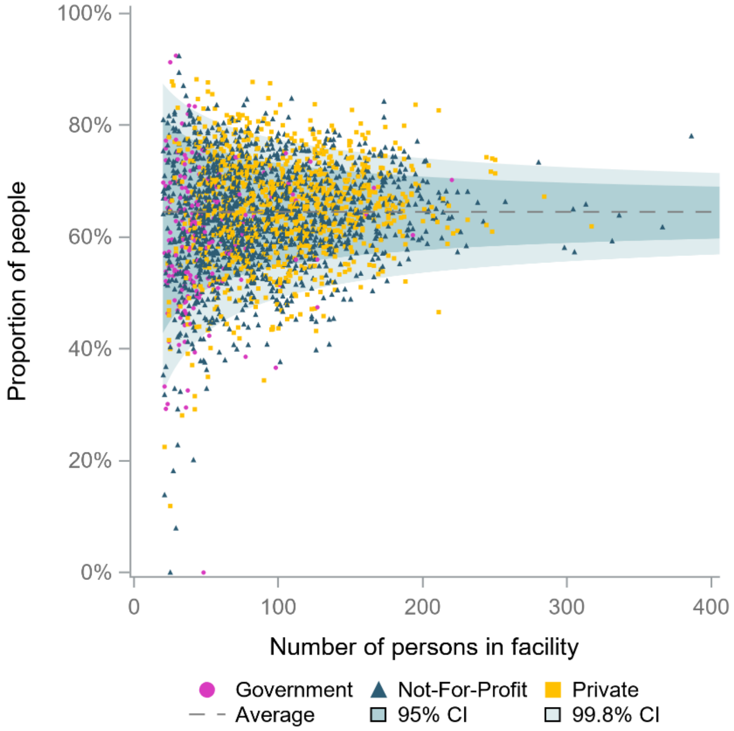

Indicator 4: Adjusted premature mortality among residents of 2638 residential aged care facilities in Australia

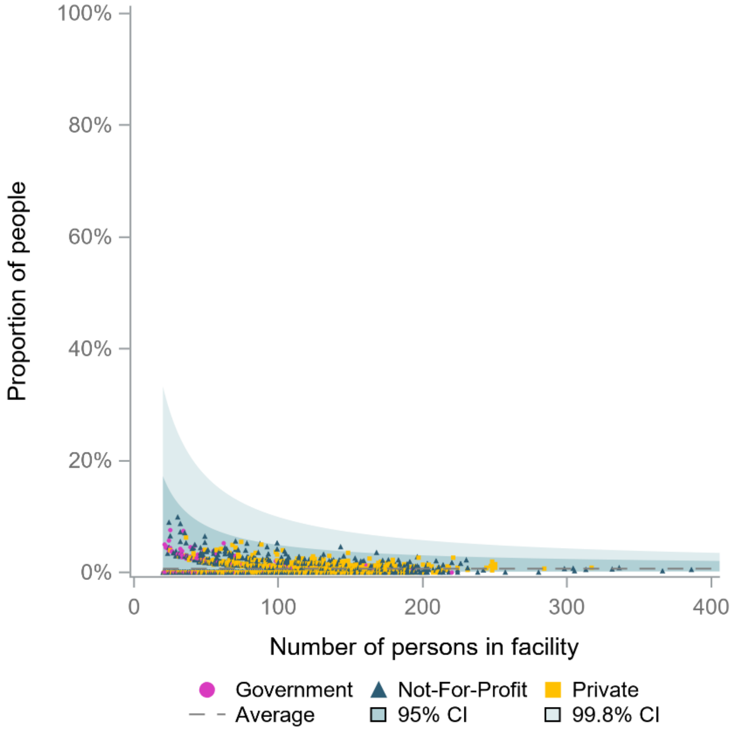

Indicator 5: Adjusted fall-related hospitalisation among residents of 2290 residential aged care facilities in four states

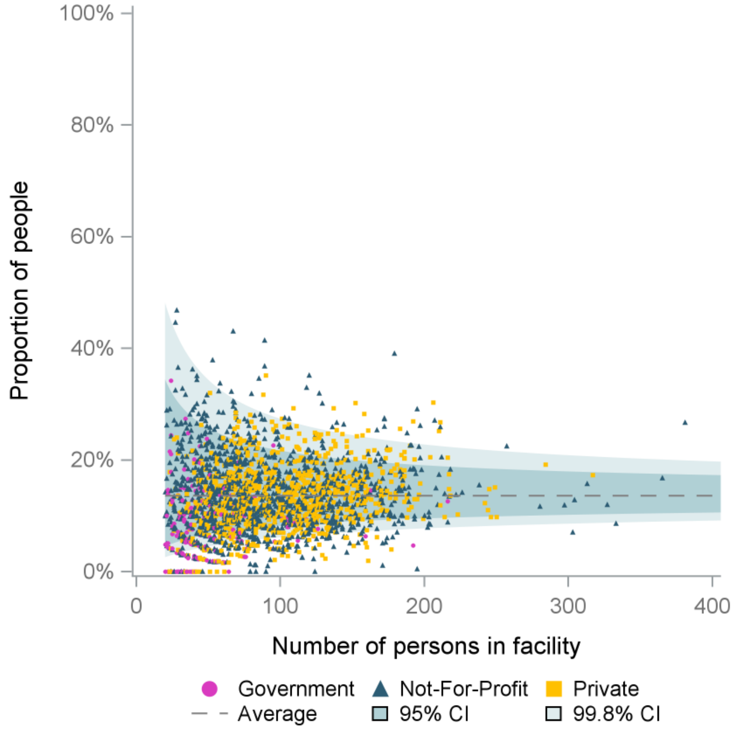

Indicator 6: Adjusted fracture among residents of 2290 residential aged care facilities in four states

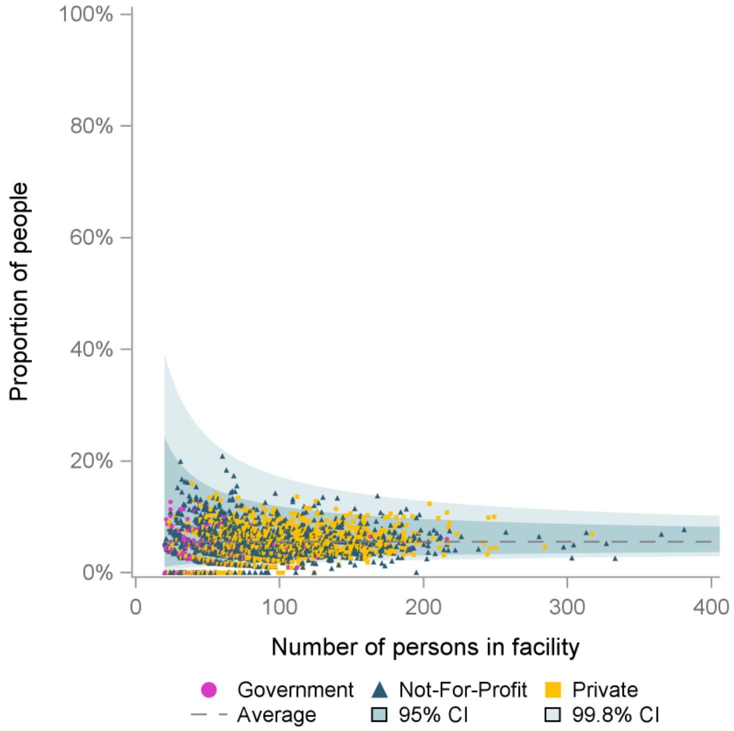

Indicator 7: Adjusted weight loss or malnutrition-related hospitalisation among residents of 2287 residential aged care facilities in four states

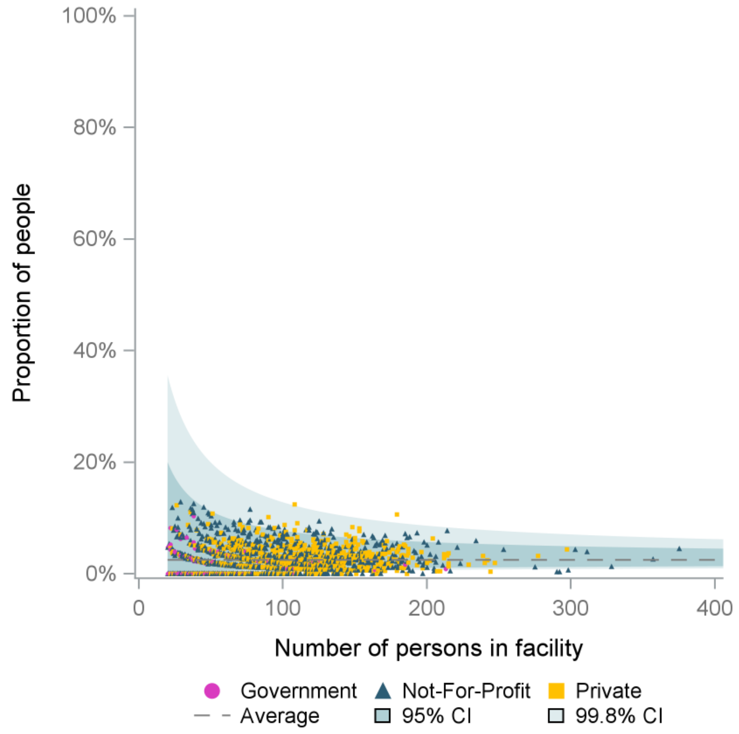

Indicator 8: Adjusted medication-related hospitalisation among residents of 2295 residential aged care facilities in four states

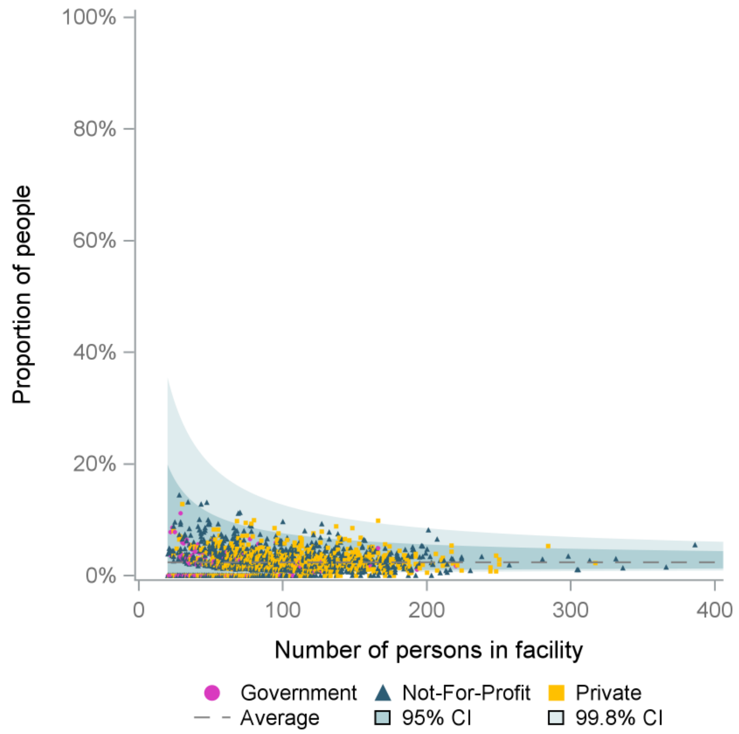

Indicator 9: Adjusted delirium or dementia-related hospitalisation among residents of 1998 residential aged care facilities in four states

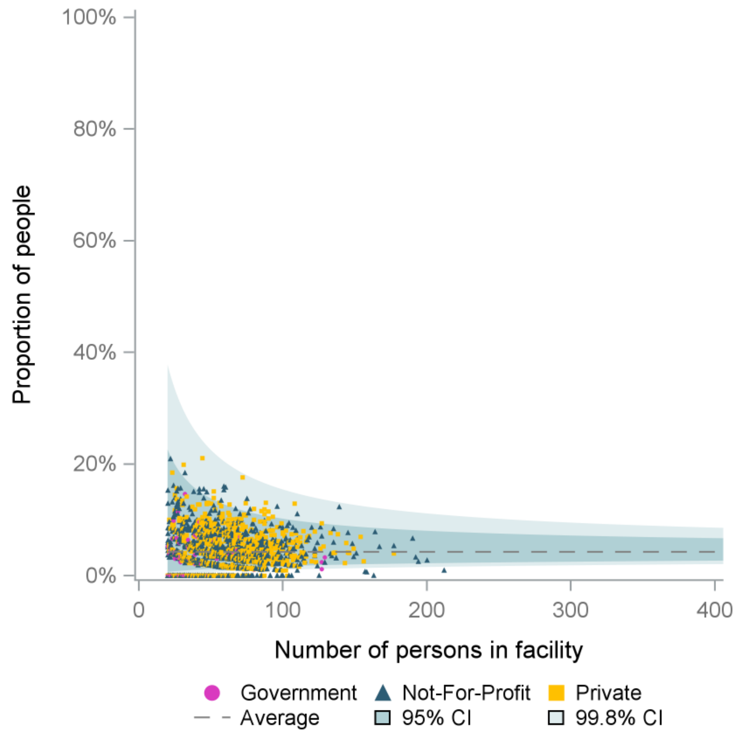

Indicator 10: Adjusted pressure injury-related hospitalisation among residents of 2295 residential aged care facilities in four states

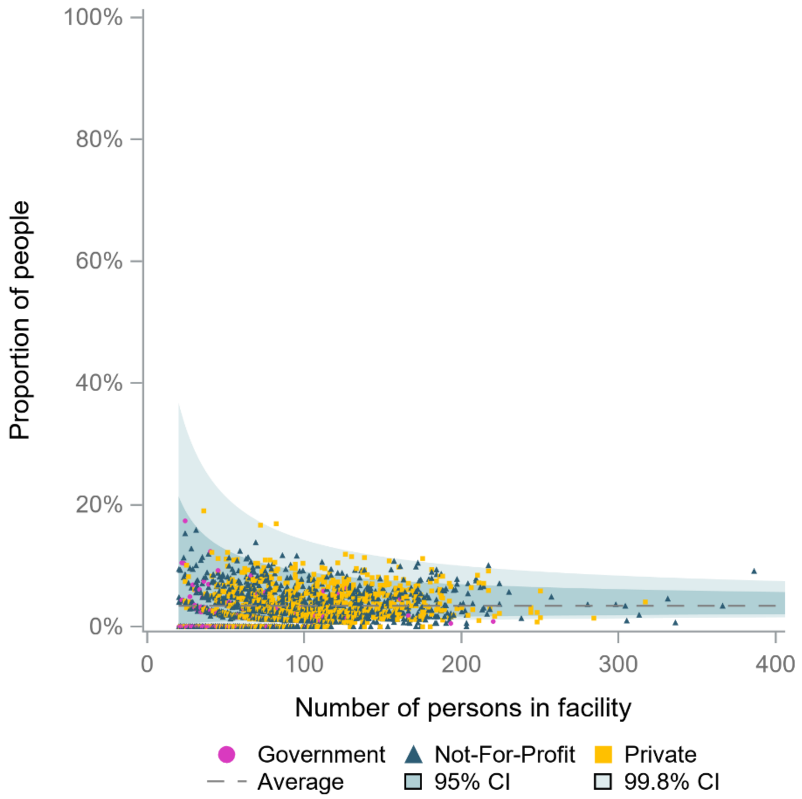

Figure 2. Funnel plots illustrating home care service level variations in care, by ownership, 2019

Indicator 1: Adjusted high sedative load among home care recipients from 1488 services in Australia

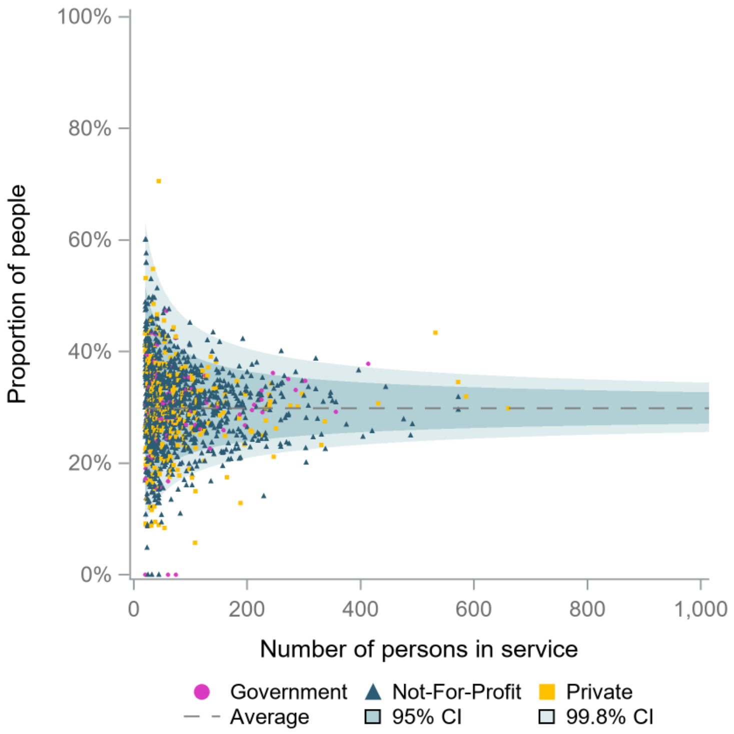

Indicator 2: Adjusted antibiotic use among home care recipients from 1507 services in Australia

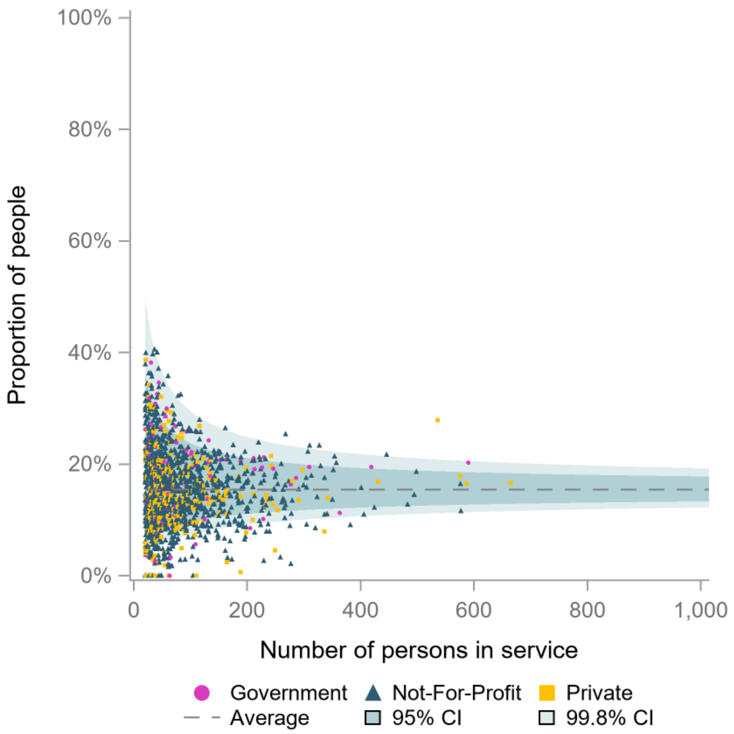

Indicator 3: Adjusted chronic opioid use among home care recipients from 1492 services in Australia

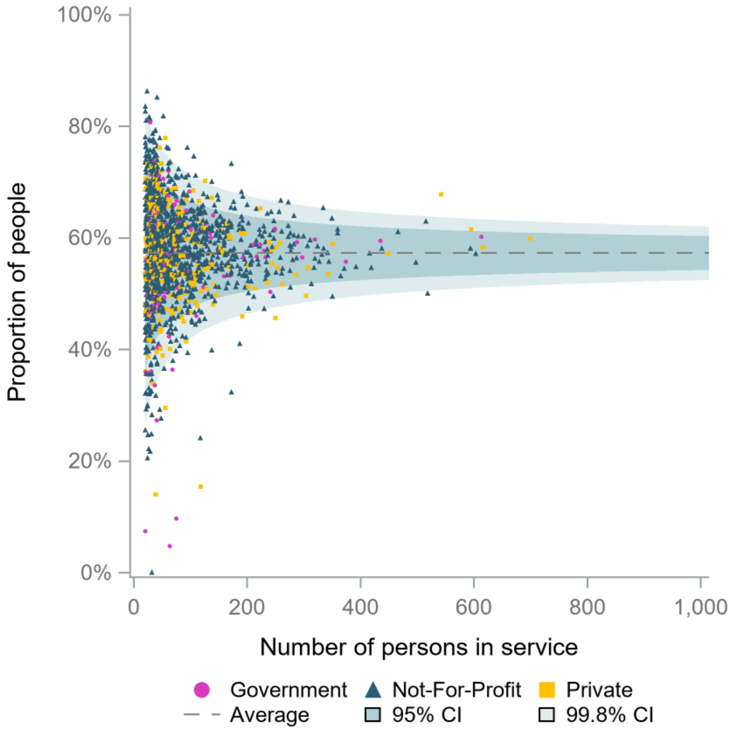

Indicator 4: Adjusted premature mortality among home care recipients from 1507 services in Australia

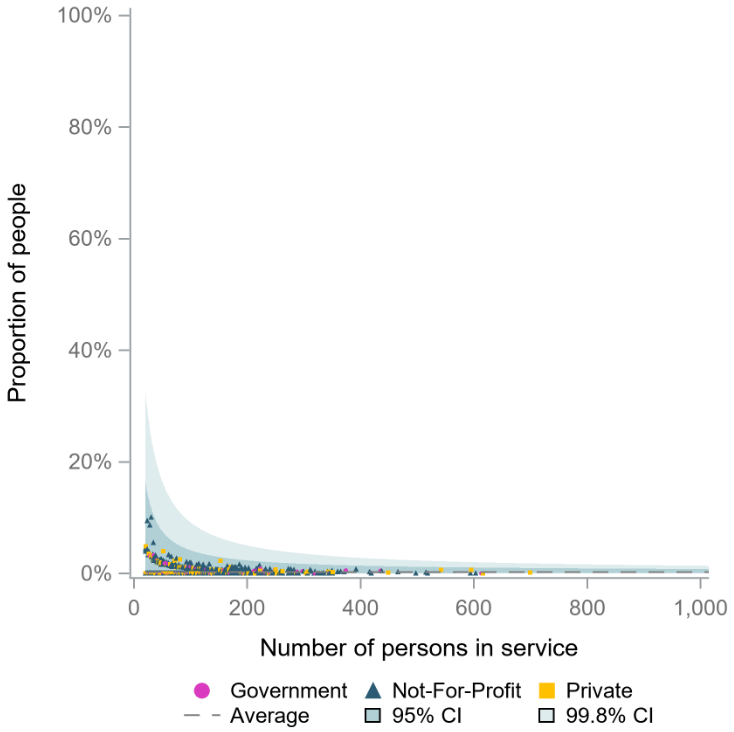

Indicator 5: Adjusted home medicine reviews among home care recipients from 1477 services in Australia

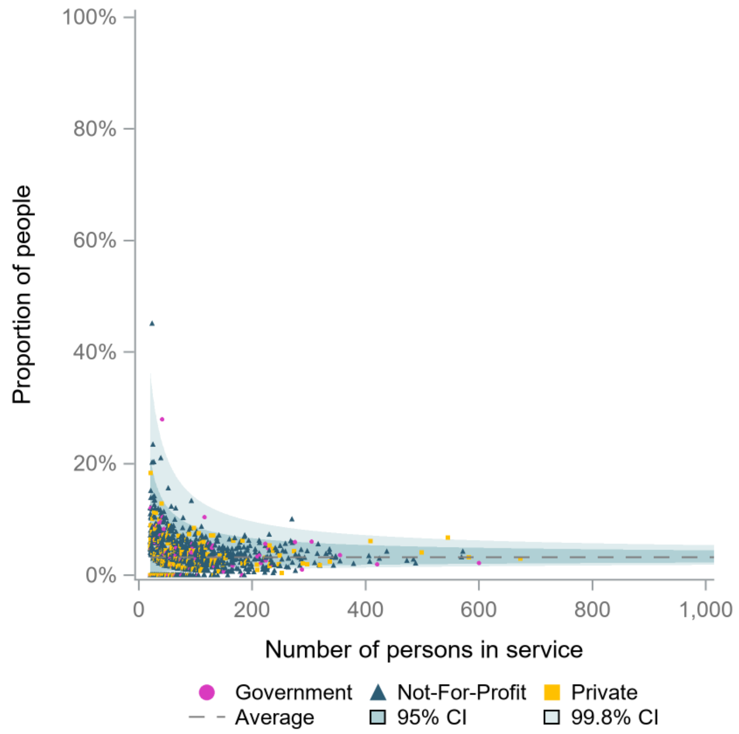

Indicator 6: Adjusted chronic disease management among home care recipients from 1388 services in Australia

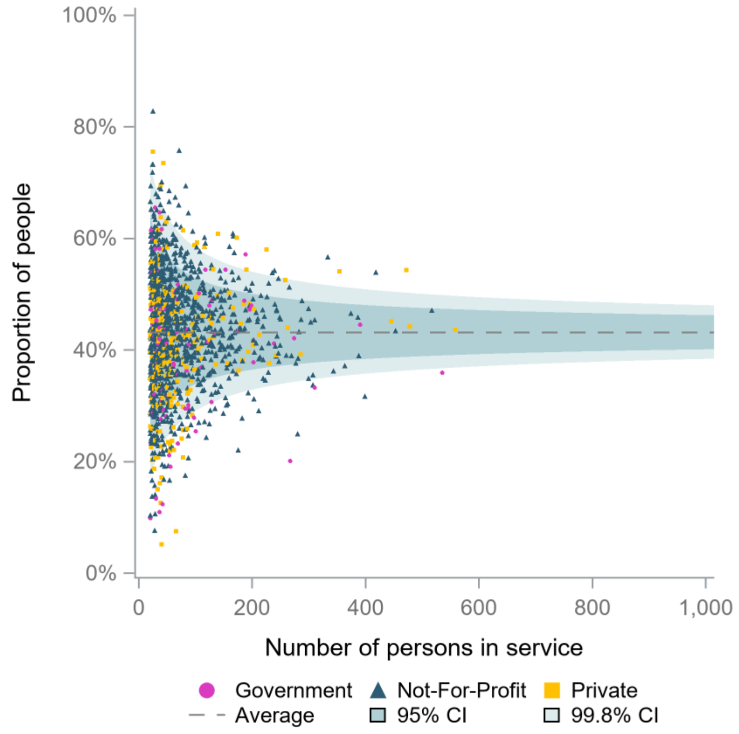

Indicator 7: Wait-time for home care services among home care recipients from 687 services in Australia

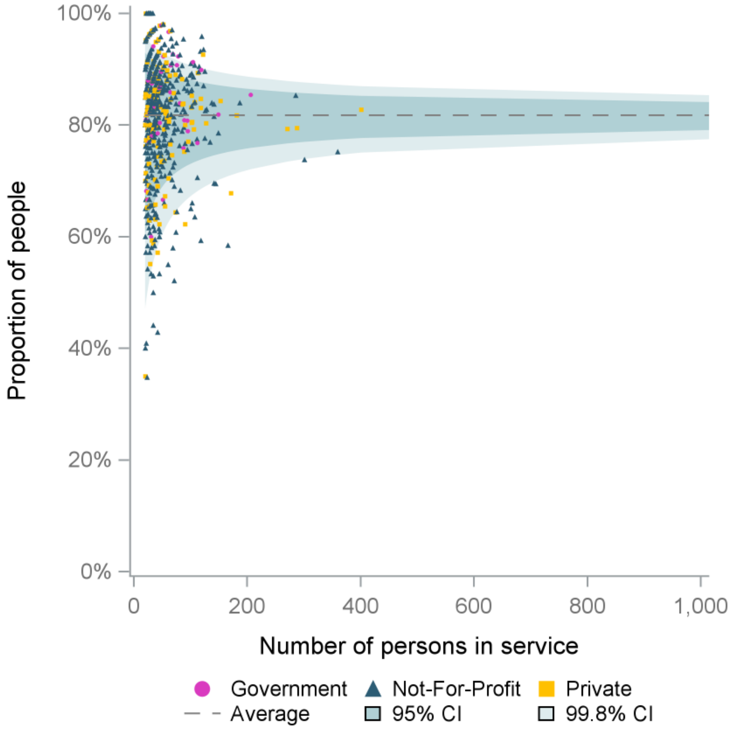

Indicator 8: Adjusted fall-related hospitalisation among home care recipients from 1276 services in four states

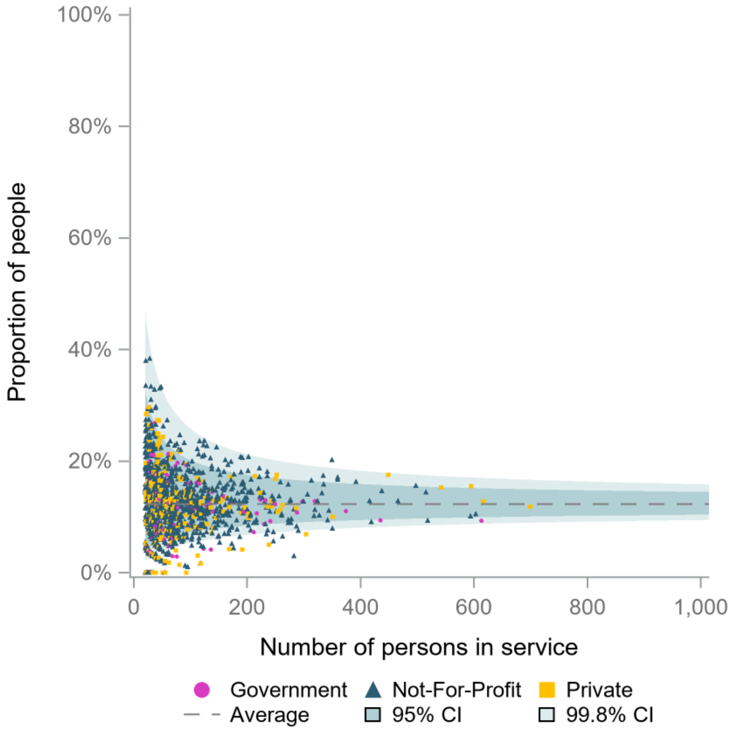

Indicator 9: Adjusted fracture among home care recipients from 1276 services in four states

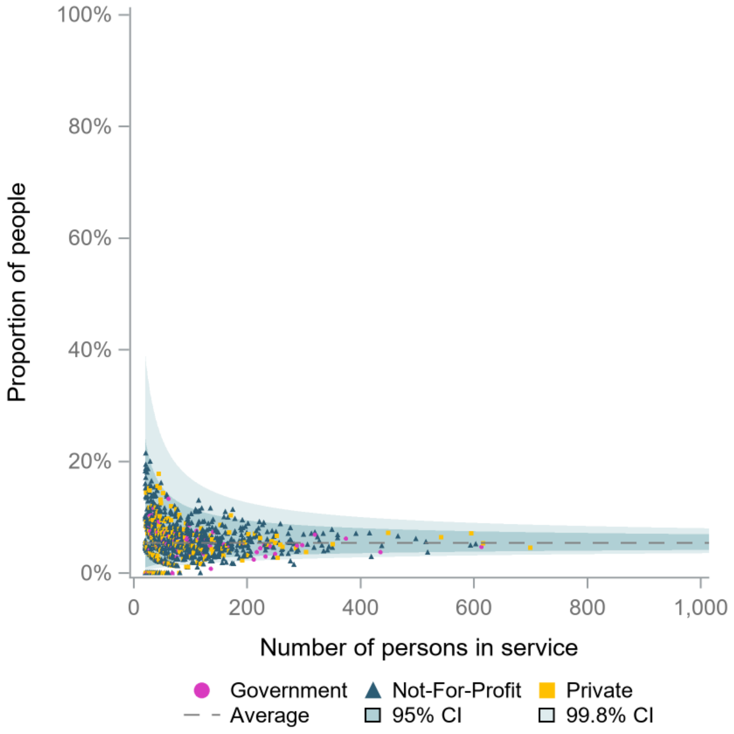

Indicator 10: Adjusted weight loss/malnutrition-related hospitalisation among home care recipients from 1264 services in four states

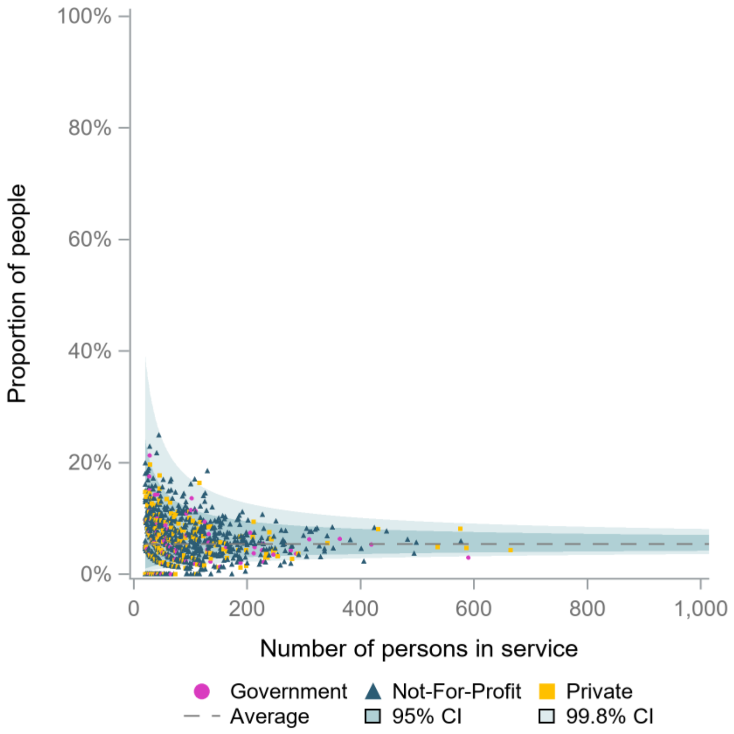

Indicator 11: Adjusted medication-related hospitalisation among home care recipients from 1276 services in four states

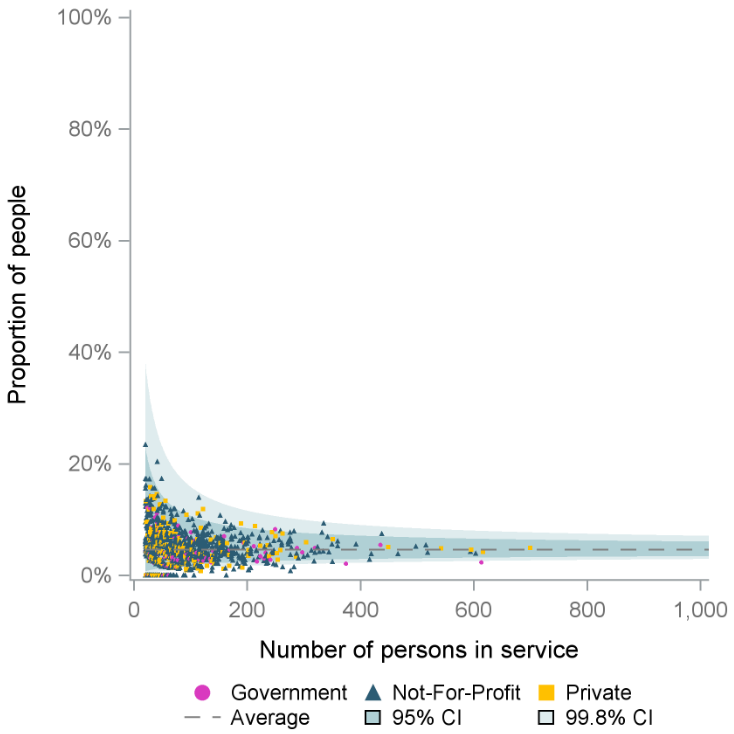

Indicator 12: Adjusted delirium/dementia-related hospitalisation among home care recipients from 350 services in four states

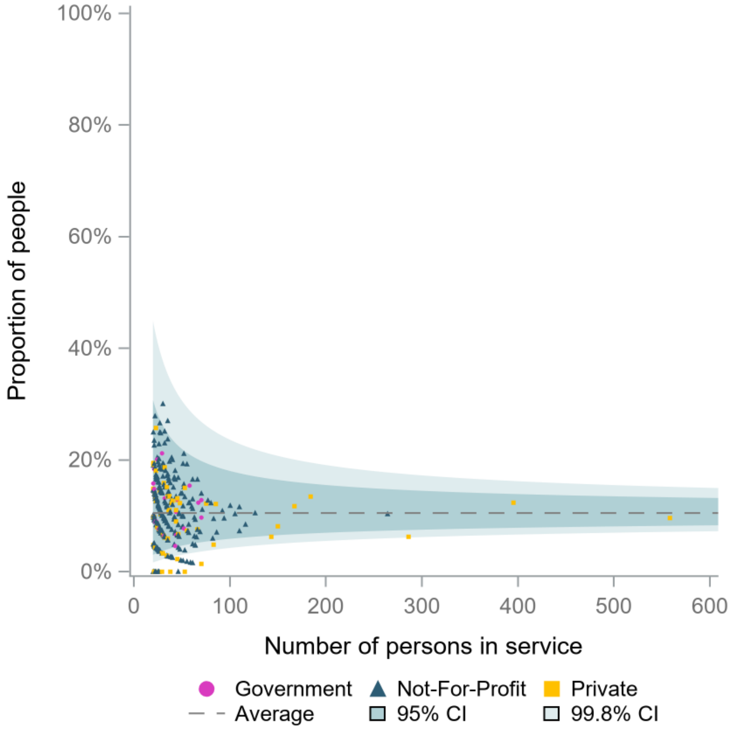

Indicator 13: Adjusted pressure injury-related hospitalisation among home care recipients from 1276 services in four states

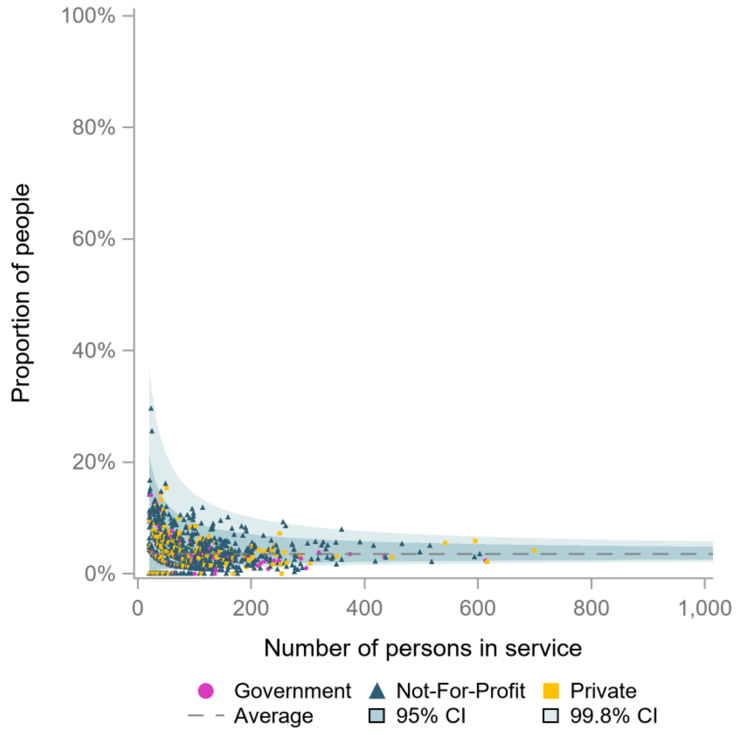

The RECORD statement – checklist of items, extended from the STROBE statement, that should be reported in observational studies using routinely collected health data.

Note: The page and section numbers in this checklist refer to the submitted manuscript, not to the published article or its Supporting Information file

	Item No.	STROBE items	Location in manuscript where items are reported	RECORD items	Location in manuscript where items are reported
**Title and abstract**					
	1	(a) Indicate the study's design with a commonly used term in the title or the abstract (b) Provide in the abstract an informative and balanced summary of what was done and what was found	Page 1	RECORD 1.1: The type of data used should be specified in the title or abstract. When possible, the name of the databases used should be included.<br><br>RECORD 1.2: If applicable, the geographic region and timeframe within which the study took place should be reported in the title or abstract.<br><br>RECORD 1.3: If linkage between databases was conducted for the study, this should be clearly stated in the title or abstract.	Page 1
**Introduction**					
Background rationale	2	Explain the scientific background and rationale for the investigation being reported	Page 5		
Objectives	3	State specific objectives, including any prespecified hypotheses	Page 5		
**Methods**					
Study Design	4	Present key elements of study design early in the paper	Page 6		
Setting	5	Describe the setting, locations, and relevant dates, including periods of recruitment, exposure, follow-up, and data collection	Page 6		
Participants	6	*(a) Cohort study* - Give the eligibility criteria, and the sources and methods of selection of participants. Describe methods of follow-up<br>*Case-control study* - Give the eligibility criteria, and the sources and methods of case ascertainment and control selection. Give the rationale for the choice of cases and controls<br>*Cross-sectional study* - Give the eligibility criteria, and the sources and methods of selection of participants	Page 5-6	RECORD 6.1: The methods of study population selection (such as codes or algorithms used to identify subjects) should be listed in detail. If this is not possible, an explanation should be provided.<br><br>RECORD 6.2: Any validation studies of the codes or algorithms used to select the population should be referenced. If validation was conducted for this study and not published elsewhere, detailed methods and results should be provided.	Page 5-6

		(b) Cohort study - For matched studies, give matching criteria and number of exposed and unexposed<br>*Case-control study* - For matched studies, give matching criteria and the number of controls per case		RECORD 6.3: If the study involved linkage of databases, consider use of a flow diagram or other graphical display to demonstrate the data linkage process, including the number of individuals with linked data at each stage.	
Variables	7	Clearly define all outcomes, exposures, predictors, potential confounders, and effect modifiers. Give diagnostic criteria, if applicable.	Page 6, Supporting Information, Table 1 and 2	RECORD 7.1: A complete list of codes and algorithms used to classify exposures, outcomes, confounders, and effect modifiers should be provided. If these cannot be reported, an explanation should be provided.	Page 6, Supporting Information, Table 1 and 2
Data sources/ measurement	8	For each variable of interest, give sources of data and details of methods of assessment (measurement). Describe comparability of assessment methods if there is more than one group	Page 5-6, Supporting Information, Table 1 and 2		
Bias	9	Describe any efforts to address potential sources of bias	Page 6		
Study size	10	Explain how the study size was arrived at	Page 6		
Quantitative variables	11	Explain how quantitative variables were handled in the analyses. If applicable, describe which groupings were chosen, and why	Page 6		
Statistical methods	12	(a) Describe all statistical methods, including those used to control for confounding<br>(b) Describe any methods used to examine subgroups and interactions<br>(c) Explain how missing data were addressed<br>(d) *Cohort study* - If applicable, explain how loss to follow-up was addressed<br>*Case-control study* - If applicable, explain how matching of cases and controls was addressed<br>*Cross-sectional study* - If applicable, describe analytical methods taking account of sampling strategy<br>(e) Describe any sensitivity analyses	Page 6		
Data access and cleaning methods		..		RECORD 12.1: Authors should describe the extent to which the investigators had	Page 5-6

				access to the database population used to create the study population.<br><br>RECORD 12.2: Authors should provide information on the data cleaning methods used in the study.	
Linkage		..		RECORD 12.3: State whether the study included person-level, institutional-level, or other data linkage across two or more databases. The methods of linkage and methods of linkage quality evaluation should be provided.	Page 5-6
**Results**					
Participants	13	(a) Report the numbers of individuals at each stage of the study (*e.g.*, numbers potentially eligible, examined for eligibility, confirmed eligible, included in the study, completing follow-up, and analysed)<br>(b) Give reasons for non-participation at each stage.<br>(c) Consider use of a flow diagram	Page 4, 6-7	RECORD 13.1: Describe in detail the selection of the persons included in the study (*i.e.*, study population selection) including filtering based on data quality, data availability and linkage. The selection of included persons can be described in the text and/or by means of the study flow diagram.	Page 7
Descriptive data	14	(a) Give characteristics of study participants (*e.g.*, demographic, clinical, social) and information on exposures and potential confounders<br>(b) Indicate the number of participants with missing data for each variable of interest<br>(c) *Cohort study* - summarise follow-up time (*e.g.*, average and total amount)	Page 7-8		
Outcome data	15	*Cohort study* - Report numbers of outcome events or summary measures over time<br>*Case-control study* - Report numbers in each exposure category, or summary measures of exposure<br>*Cross-sectional study* - Report numbers of outcome events or summary measures	Page 7-8, Supporting Information, Tables 3-4 and Figure 1 and 2		
Main results	16	(a) Give unadjusted estimates and, if applicable, confounder-adjusted estimates and their precision (e.g., 95% confidence interval). Make clear which confounders were	Page 7-8		

		adjusted for and why they were included<br>(b) Report category boundaries when continuous variables were categorized<br>(c) If relevant, consider translating estimates of relative risk into absolute risk for a meaningful time period			
Other analyses	17	Report other analyses done—e.g., analyses of subgroups and interactions, and sensitivity analyses			
**Discussion**					
Key results	18	Summarise key results with reference to study objectives	Page 8		
Limitations	19	Discuss limitations of the study, taking into account sources of potential bias or imprecision. Discuss both direction and magnitude of any potential bias	Page 9	RECORD 19.1: Discuss the implications of using data that were not created or collected to answer the specific research question(s). Include discussion of misclassification bias, unmeasured confounding, missing data, and changing eligibility over time, as they pertain to the study being reported.	Page 9
Interpretation	20	Give a cautious overall interpretation of results considering objectives, limitations, multiplicity of analyses, results from similar studies, and other relevant evidence	Page 8-10		
Generalisability	21	Discuss the generalisability (external validity) of the study results	Page 9		
**Other Information**					
Funding	22	Give the source of funding and the role of the funders for the present study and, if applicable, for the original study on which the present article is based			
Accessibility of protocol, raw data, and programming code		..		RECORD 22.1: Authors should provide information on how to access any supplemental information such as the study protocol, raw data, or programming code.	

*Reference: Benchimol EI, Smeeth L, Guttmann A, Harron K, Moher D, Petersen I, Sørensen HT, von Elm E, Langan SM, the RECORD Working Committee. The REporting of studies Conducted using Observational Routinely-collected health Data (RECORD) Statement. *PLoS Medicine* 2015; in press.

*Checklist is protected under Creative Commons Attribution (CC BY) license.
